# ADAD2 regulates heterochromatin in meiotic and post-meiotic male germ cells via translation of MDC1

**DOI:** 10.1242/jcs.259196

**Published:** 2022-02-22

**Authors:** Lauren G. Chukrallah, Aditi Badrinath, Gabrielle G. Vittor, Elizabeth M. Snyder

**Affiliations:** Department of Animal Science, Rutgers University, New Brunswick, NJ 08901, USA

**Keywords:** Chromatin, Germ cells, Meiosis, RNA-binding proteins, mRNA translation, Spermatogenesis

## Abstract

Male germ cells establish a unique heterochromatin domain, the XY-body, early in meiosis. How this domain is maintained through the end of meiosis and into post-meiotic germ cell differentiation is poorly understood. ADAD2 is a late meiotic male germ cell-specific RNA-binding protein, loss of which leads to post-meiotic germ cell defects. Analysis of ribosome association in *Adad2* mouse mutants revealed defective translation of *Mdc1*, a key regulator of XY-body formation, late in meiosis. As a result, *Adad2* mutants show normal establishment but failed maintenance of the XY-body. Observed XY-body defects are concurrent with abnormal autosomal heterochromatin and ultimately lead to severely perturbed post-meiotic germ cell heterochromatin and cell death. These findings highlight the requirement of ADAD2 for *Mdc1* translation, the role of MDC1 in maintaining meiotic male germ cell heterochromatin and the importance of late meiotic heterochromatin for normal post-meiotic germ cell differentiation.

## Teaser

**Summary:** ADAD2 is required for MDC1 translation, the reduction of which leads to abnormal heterochromatin and post-meiotic cell death, and is a key heterochromatin maintenance factor in male germ cells.

## INTRODUCTION

Male germ cell differentiation is accompanied by radical changes in the chromatin landscape as cells progress from mitosis to meiosis to spermiogenesis (Kota and Feil, 2010). Of particular importance is the formation early in meiosis of the XY-body, a heterochromatic region encapsulating the X and Y chromosome. Failure to form an XY-body results in meiotic cell arrest and infertility (Abe et al., 2020; Fernandez-Capetillo et al., 2003; Ichijima et al., 2011). Thus, although factors initiating this event have been well described, how the unique chromatin state of the XY-body is maintained through the later stages of meiosis and whether this is important for post-meiotic steps of male germ cell differentiation is unknown.

In early meiotic spermatocytes, pericentric heterochromatin (PCH) of both sex chromosomes and autosomes is composed of similar epigenetic marks (Magaraki et al., 2017). However, with the onset of chromosome synapsis, PCH and other heterochromatin marks diverge between the sex chromosomes and the autosomes, resulting in two unique chromatin compartments, autosomal PCH and the XY-body, composed of sex chromosome PCH and other sex-chromosome-enriched epigenetic marks (Namekawa et al., 2006). These compartments ultimately give rise to distinct compartments in the post-meiotic germ cell, with autosomal PCH forming a region termed the chromocenter (CC) and sex chromosome PCH forming post-meiotic sex chromatin (PMSC) (Haaf and Ward, 1995; Namekawa et al., 2006). Although genetic evidence demonstrates XY-body formation is required in early and mid-meiosis to suppress synapsis checkpoints and silence sex chromosome gene expression (meiotic sex chromosome inactivation – MSCI) (McKee and Handel, 1993), it is unknown whether stringent maintenance in late meiosis is necessary for post-meiotic germ cell differentiation and how maintenance may be achieved.

Two key mediators of XY-body formation are breast cancer 1 (BRCA1) and mediator of DNA-damage checkpoint protein 1 (MDC1). In male germ cells, BRCA1 is required for the initial establishment of X-chromosome γH2AX through its recruitment of ATR and TOPBP1, ultimately forming X-chromosome PCH (Broering et al., 2014). MDC1, on the other hand, interacts with γH2AX (Ichijima et al., 2011; Savic et al., 2009) and leads to spreading of the γH2AX mark throughout the X and Y, giving rise to the XY-body (Ichijima et al., 2011; Namekawa et al., 2006). Although the MDC1-γH2AX pathway has been traditionally associated with the DNA damage response (DDR) (Blanco-Rodriguez, 2012; Savic et al., 2009) and in recombination via regulation of MLH3 (Testa et al., 2018), a growing consensus contends that meiotic male germ cells leverage this pathway to facilitate chromatin-induced silencing of the X and Y chromosome early in meiosis. In support of this, MDC1 either directly or indirectly influences the XY-body localization of multiple chromatin remodeling proteins required to establish the unique epigenetic signature of the XY-body (Ichijima et al., 2011). MDC1 and BRCA1 remain high throughout meiosis (Ahmed et al., 2007; Turner et al., 2004), suggesting they may be important in the maintenance of the XY-body; however, mutation of either results in germ cell loss prior to meiotic completion (Ahmed et al., 2007), thus their roles in late meiosis and post-meiotic events are unclear.

Several lines of evidence suggest the chromatin state of the XY-body late in meiosis is important for post-meiotic germ cell development. The chromatin composition of the CC and PMSC closely mimics their meiotic counterparts. Furthermore, mutation of the MDC1-interacting protein Scm polycomb group protein like 2 (SCML2), leads to dysregulation of spermatid chromatin (Luo et al., 2015). Mutation of a second meiotic chromatin remodeling protein, bromodomain testis associated (BRDT), alters heterochromatin in both meiotic compartments during late meiosis and displays distinct CC abnormalities coupled with defects in sperm maturation (Berkovits and Wolgemuth, 2011). As spermatid chromatin state is thought to establish the nuclear topology necessary for genome compaction in the sperm head (Haaf and Ward, 1995; Meyer-Ficca et al., 1998), these observations couple meiotic chromatin state with final germ cell genome remodeling. The underlying mechanisms driving this phenomenon are, as yet, unknown.

Translation regulation, which is mediated by RNA-binding proteins (RBPs), is a hallmark of meiotic and post-meiotic male germ cells (Braun et al., 1989; Kleene et al., 1984). As a result, there is a solid understanding of how RBPs facilitate translation repression during meiosis (Yang et al., 2007). Although there has been no equivalent study of how RBPs enhance translation during meiosis and the physiological importance thereof, genetic models have shown that abnormal translation initiation can lead to failed meiotic progression (Sun et al., 2010). Furthermore, translation activation during meiosis is dynamically regulated in both mammalian oocytes (Susor et al., 2016) and yeast (Brar et al., 2012). Coupled to this, male germ cells face the additional complexity arising from MSCI that prevents expression of X-chromosome genes, such as *Scml2*, late in meiosis (Mueller et al., 2008) where they are required for meiotic events (Hasegawa et al., 2015). Together, these observations suggest positive regulation of translation may be an important regulator of male germ cell meiosis; however, the relevant RBPs, their mRNA targets and the physiological outcome of abnormal translation have yet to be elucidated.

ADAD2 is a germ cell-specific RBP necessary for post-meiotic germ cell differentiation; *Adad2* mutant germ cells fail to undergo the final stage of development, spermiogenesis (Chukrallah et al., 2020). However, the molecular underpinnings of germ cell loss in the *Adad2* mutant are unknown. Paradoxically, ADAD2 is expressed and detected exclusively in mid- and late meiotic germ cells, suggesting it regulates processes in meiosis that are important for post-meiotic events (Chukrallah et al., 2020). One of the key events of post-meiotic differentiation is the establishment of a compacted haploid genome via the sequential replacement of histones first with transition proteins followed by protamines (Braun, 2001; Yu et al., 2000; Zhao et al., 2004). However, it remains unclear whether epigenetic programming in the meiotic cell directly influences post-meiotic chromatin transitions.

Here, we demonstrate ADAD2 is required for normal translation of *Mdc1* late in meiosis. In *Adad2* mutants, defective *Mdc1* translation gives rise to aberrant heterochromatin in both autosomes and the sex chromosomes of late meiotic spermatocytes. These defects are retained in haploid spermatids, which ultimately undergo arrest and apoptosis. Our studies define the mechanism of germ cell death in *Adad2* mutants and highlight the central role of MDC1 in maintaining heterochromatin in both chromatin compartments of the late meiotic germ cell, the importance of this maintenance for normal post-meiotic germ cell chromatin and the key role of translation regulation therein.

## RESULTS

### *Adad2* mutant round spermatids exhibit abnormal chromatin structure

**Fig. 1.**
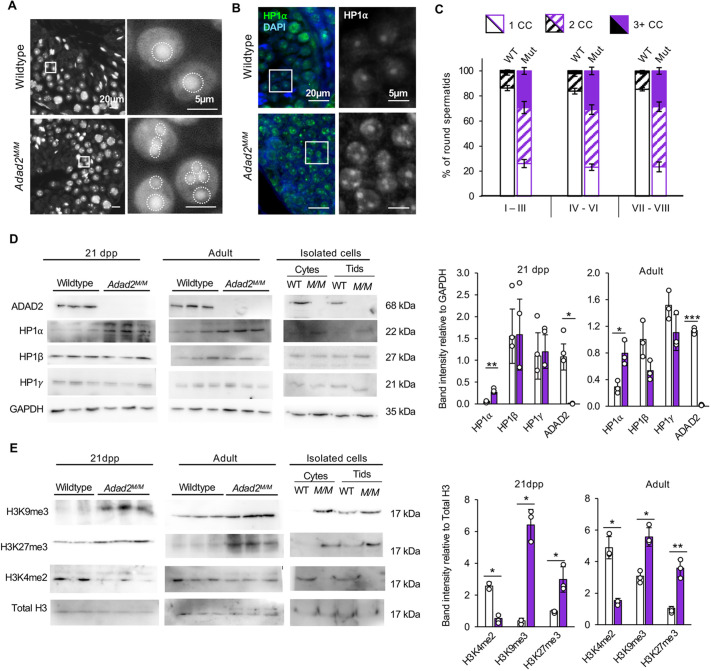
***Adad2* mutant meiotic spermatocytes and post-meiotic spermatids have abnormal localization and increased levels of heterochromatin.** (A) DAPI-stained adult wild-type and *Adad2* mutant (*Adad2^M/M^*) stage-matched tubules show a single DAPI-intense focus (chromocenter) in wild-type round spermatids and an increased number of DAPI-intense foci in mutant round spermatids. The area outlined in the images on the left is shown in more detail on the right. Dashed circles indicate DAPI-intense foci. Quantification of DAPI-intense foci demonstrates a significant increase in mutant spermatids. (B) Immunofluorescence of HP1α in wild-type and *Adad2* 21 dpp testis reveals a single HP1α focus in wild-type spermatids and multiple foci in mutant spermatids, consistent with abnormal chromocenter number in mutants. The area outlined in the images on the left is shown in more detail on the right. Green indicates HP1α, blue indicates DAPI. HP1α only is shown on the right. (C) Chromocenter number by stage in adult wild-type and *Adad2^M/M^* round spermatids (three biological samples per genotype) demonstrating high chromocenter number throughout development in mutant cells. Error bars indicate s.d. (D) Western blot of HP1 proteins in 21 dpp and adult whole-testis lysate from wild-type and *Adad2^M/M^* samples (three biological samples per genotype) and from enriched pools of adult wild-type and *Adad2^M/M^* spermatocytes and spermatids (pooled across three biological samples per genotype). ADAD2 and GAPDH are genotype and loading controls, respectively. The 21 dpp ADAD2 genotype control and GAPDH loading control blots are also shown for the same protein panel in Fig. 3A, Fig. S5B,D and Fig. S6D. The adult ADAD2 genotype control and GAPDH loading control blots are also shown for the same protein sample panel in Fig. S5D. Quantification of band intensity confirms a significant increase of HP1α in mutants at both ages. (E) Western blot of select epigenetic marks in 21 dpp and adult whole-testis histone lysate from wild-type and *Adad2^M/M^ *samples (*n*=2 samples for 21 dpp wild type; *n*=3 for all others) and enriched pools of adult wild-type and *Adad2^M/M^* spermatocytes and spermatids (pooled across three biological samples per genotype) with total histone 3 (H3) as a loading control. The 21 dpp total H3 loading control blot is also shown for the same protein sample panel in Fig. S6A. Quantification of band intensity shows increased heterochromatin, measured by H3K9me3 and H3K27me3, and decreased euchromatin, measured by H3K4me2. Throughout, dots represent individual samples; data are mean±s.d. Significance was calculated using an unpaired, two-tailed Student‘s *t-*test (*P<0.05, **P<0.001, ***P<0.0001).

*Adad2* mutants (*Adad2^em3^*, referred to herein as *Adad2^M/M^*) display post-meiotic germ cell loss (Chukrallah et al., 2020). In our effort to understand the molecular underpinnings of the *Adad2^M/M^* phenotype, we examined post-meiotic germ cell morphology in *Adad2^M/M^* testis. The chromocenter, a hallmark of post-meiotic round spermatids, is a heterochromatic region central to spermatid nuclear organization (Berkovits and Wolgemuth, 2011) easily assessed via DAPI staining. As expected, this analysis (Fig. 1A) showed wild-type round spermatids nearly always contained a single, well-defined chromocenter. In contrast, *Adad2^M/M^* round spermatids contained multiple regions of dense DAPI staining, often displaying dramatic increases in chromocenter-like foci relative to wild type. We confirmed the heterochromatic nature of these morphological features via immunofluorescent staining with the heterochromatin mark HP1α (Fig. 1B).

To determine when, during round spermatid development, chromocenter number increased in *Adad2* mutants, we quantified chromocenter number per round spermatid as a function of stage (Fig. 1C). As *Adad2* mutant tubules do not contain the full complement of post-meiotic cells normally used for staging, we relied instead on the immunofluorescent staining pattern of SYCP3 to aid in stage identification (Fig. S1A). This analysis demonstrated chromocenter defects in the earliest population of round spermatids that did not increase through round spermatid development, suggesting heterochromatin abnormalities in mutant spermatids arise prior to post-meiotic differentiation.

Last, we quantified mutant spermatid apoptosis via TUNEL (Fig. S2A,B). For round spermatids in stages I through VIII, we observed a bimodal distribution with roughly half of the mutant tubules containing many TUNEL-positive cells and the remaining having none, suggesting spermatid apoptosis occurred in distinct spermatid populations. To define which population was undergoing apoptosis, the number of round spermatids per tubule were quantified as a function of stage in adult testes (Fig. S2C). This analysis demonstrated an early (stage I-III) reduction of round spermatids in mutant testes relative to wild type, which held constant throughout the remainder of development. Together, these results imply an early loss of round spermatids at or just after the transition out of meiosis. This conclusion is further supported by normal levels of TUNEL-positive spermatocytes in mutant adults (Fig. S2D), which confirmed increased apoptotic germ cells were limited to post-meiotic spermatids.

### Abnormal chromatin structure in *Adad2* mutant spermatids is a result of increased heterochromatin

The increase in chromocenter number observed in *Adad2^M/M^* may be a result of abnormal heterochromatin condensation or an overall heterochromatin increase. To distinguish between these two, we quantified the abundance of three chromobox (CBX) proteins (referred to herein as HP1 proteins: HP1α, HP1β and HP1γ), which are well-characterized markers of heterochromatin with distinct expression and localization patterns in meiotic and post-meiotic germ cells (Brown et al., 2010; Charaka et al., 2020; Chevillard et al., 1993) (Fig. 1D). The HP1 proteins were examined at two points in testis development: the adult, which contains the full complement of germ cells and is dominated by post-meiotic germ cells; and 21 dpp, in which late meiotic germ cells are the most abundant cell population, to estimate when heterochromatin changes may arise in the mutant. Of the three HP1 proteins, only HP1α was found to be significantly increased, with this increase most apparent at 21 dpp. Although HP1β and HP1γ are expressed early in the pachytene phase of meiosis, HP1α functions much later in meiosis (Takada et al., 2011). Thus, the increase of HP1α, but not HP1β or HP1γ, suggests increased heterochromatin in the *Adad2* mutants arises late in pachytene, timing that coincides with expression of ADAD2 in wild-type spermatocytes. To further confirm these findings, we isolated wild-type and mutant spermatocytes and round spermatids, and examined HP1 abundance (Fig. 1D) as a function of cell type. As expected from the whole-testis analysis, mutant spermatocytes expressed higher levels of HP1α but not HP1β or HP1γ. Given the relatively high level of cell-enrichment generated by our isolation procedure (Fig. S1B), we conclude increased heterochromatin arises during spermatocyte development in mutant testes.

Although HP1α interacts with a wide range of DNA-associated proteins, it shows special affinity for K9-methylated histone H3 (H3K9me3) (Bannister et al., 2001). Thus, we examined H3K9me3 along with other epigenetic marks representative of heterochromatin (H3K27me3) and euchromatin (H3K4me2) by western blot of 21 dpp and adult testis histone lysate (Fig. 1E). At 21 dpp*, Adad2^M/M^* testes exhibited increased levels of both H3K9me3 and H3K27me3, along with reduced levels of H3K4me2, as did isolated spermatocytes (Fig. 1E), consistent with the hypothesis that *Adad2^M/M^* spermatocytes house increased heterochromatin with a subsequent reduction in euchromatin. These differences persisted into adulthood and were also observed in isolated round spermatids, demonstrating altered chromatin state was maintained in mutant germ cells beyond pachytene spermatocytes. In total, these findings suggest that mutant round spermatids may have an abnormal chromatin state, which could cause their loss in the *Adad2* mutant, and this abnormal state arises in late meiotic spermatocytes.

### *Adad2* mutation alters transcript abundance and ribosome association of specific transcripts

**Fig. 2.**
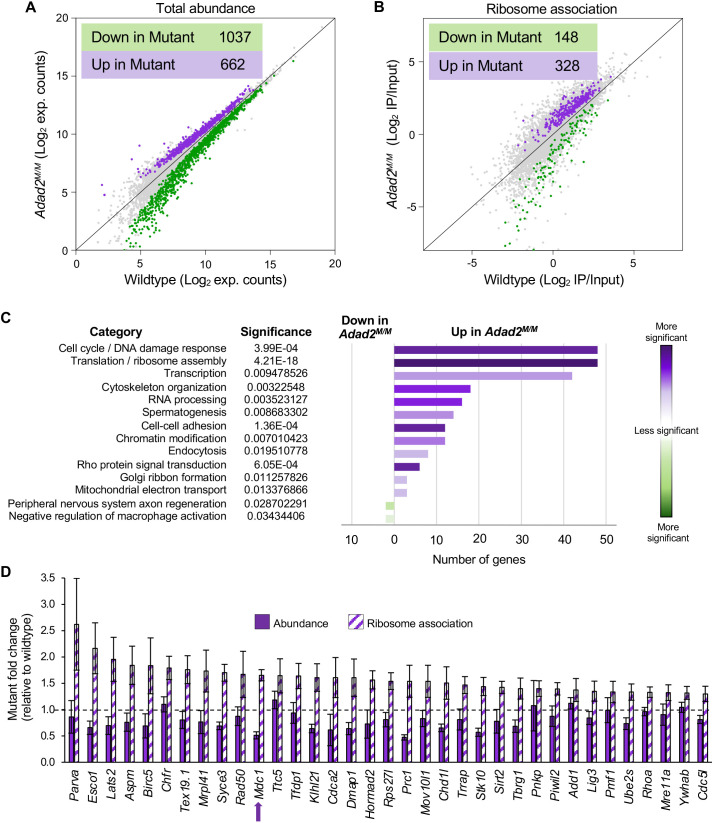
**Mutation of *Adad2* results in altered transcript abundance and ribosome association of specific transcript classes.** (A,B) Comparison of (A) total transcript abundance and (B) ribosome association in wild-type and *Adad2^M/M^ *Ribotag samples (green, downregulated in mutant; purple, upregulated in mutant) identifies many transcripts with differential ribosome association (DRA) in *Adad2* mutant testes. (C) Significantly enriched ontological categories for DRA transcripts identifies cell cycle and DNA damage response (DDR) as significantly enriched in transcripts with increased ribosome association in the mutant. (D) Total abundance and ribosome association (derived from RNA-sequencing) relative to wild type of select cell cycle and DDR-related genes identified as DRA confirms increased ribosome association and minimal abundance changes in mutant testes. Dashed line represents wild-type mean. Arrow indicates the gene of interest, *Mdc1.* Data are mean±s.d.

ADAD2 protein is exclusive to mid- and late-spermatocytes, and mutants display heterochromatin defects late in meiosis, suggesting the mutant phenotype is due to events in mid- to late meiosis. To better define causes leading to the heterochromatin defect in *Adad2* mutants, we performed RNA-sequencing of total RNA from 21 dpp wild-type and mutant testes, which are enriched for late meiotic germ cells. This analysis (Fig. 2A) identified a moderate number of differentially expressed (DE) genes (Table S2), with the majority having reduced abundance in the mutant testes (61%). Previous work examining *Adad2^M/M^* 25 dpp testes demonstrated a robust reduction in transcript abundance, most likely due to post-meiotic germ cell loss (Chukrallah et al., 2020). To determine whether this was also the case at 21 dpp, expression of DE genes in wild-type testicular somatic and germ cells was examined (Fig. S3A). This analysis showed DE transcripts reduced in the mutant were primarily expressed in wild-type meiotic and post-meiotic germ cells. Concurrently, DE transcripts increased in the mutant generally had high expression in mitotic wild-type cells, a pattern consistent with reduced numbers of meiotic and post-meiotic germ cells in the mutant. However, detailed morphological quantification of meiotic and post-meiotic cell populations (Fig. S3B,C) showed no cell loss in the mutant until the round spermatid stage, consistent with our previous TUNEL analysis (Fig. S2A,B).

Toward defining the mechanisms giving rise to the *Adad2* mutant phenotype, we examined the functions of differentially expressed genes via ontological analysis (Fig. S3D). However, of the differentially expressed genes, only 4.28% exhibited a fold-change of two-fold or higher (Table S2), demonstrating abundance changes in the mutant are relatively few in number and also of low magnitude. Ontology analysis demonstrated that genes associated with spermiogenesis and cell projection were significantly enriched in the wild-type testes, consistent with a loss of post-meiotic germ cells in the mutant. Contrary to expectation, no dramatic changes in transcripts encoding chromatin remodeling proteins or histones were observed. Together, these observations demonstrate ADAD2 loss results in a slight reduction of meiotic germ cell transcript abundance in the absence of appreciable meiotic germ cell loss and transcript abundance changes alone cannot explain the observed heterochromatin defects.

Germ cell RNA granules are known sites of translational regulation (Lehtiniemi and Kotaja, 2018) and given the previously reported granular localization of ADAD2 in meiotic germ cells (Chukrallah et al., 2020), along with its relatively limited impacts on transcript abundance, we hypothesized ADAD2 may play a role in post-transcriptional regulation. To determine the impact of *Adad2* mutation on translation, we combined our *Adad2* mutant allele with the RiboTag model (Sanz et al., 2009), which expresses a cell-specific HA-tagged large ribosomal subunit protein when driven by a cell-specific Cre. In our model, RiboTag expression was driven in differentiating germ cells via *Stra8-iCre* (*RiboTag+:Stra8-iCre+*) (Fig. S4A). Thus, RNA-sequencing of total RNA (input) and HA immunoprecipitated (IP) ribosome-RNA complexes from wild-type and mutant *RiboTag+:Stra8-iCre+* 21 dpp testes was used to quantify ribosome association across wild-type and *Adad2* mutant differentiating germ cell transcriptomes (Fig. S4B). Ribosome association (RA) was expressed as a ratio of IP over input abundance (IP/Input) to correct for any genotype-driven differential transcript abundances. From this value, differential ribosome association (DRA) was determined (Fig. 2B and listed in Table S1) and most commonly showed increased ribosome association in the mutant when compared with the wild type (69% of transcripts). Analysis of total abundance for all DRA transcripts (Fig. S4C) confirmed this was not due to overall reduced transcript abundance in the mutant testis. Furthermore, transcripts with higher RA in mutants showed the highest expression in wild-type meiotic germ cells (Fig. S4D). Together, these results suggest *Adad2* mutation results in increased ribosome association of meiotic germ cell transcripts, in agreement with the spermatocyte-specific expression of the ADAD2 protein.

We next performed ontological analysis of DRA transcripts to determine whether differential ribosome association may lead to the *Adad2^M/M^* phenotype (Fig. 2C). Although wild-type-increased DRA transcripts showed poor ontological enrichment, mutant-increased DRA transcripts had striking enrichment for translation and cell cycle functions, specifically meiosis and DNA damage response, and included many of the most profoundly impacted DRA genes (Table S1). It has been well described that meiotic male germ cells leverage the DNA damage response (DDR) pathway to establish the correct epigenetic state during meiosis (Namekawa et al., 2006); thus, we focused on defining the exact impact of differential ribosome association on select DDR proteins.

### Increased ribosome association in *Adad2* mutants results in decreased protein abundance

**Fig. 3.**
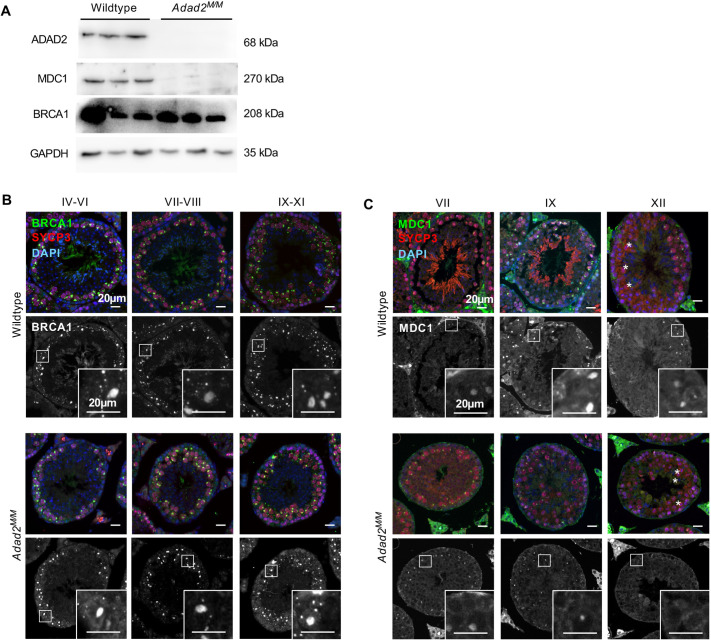
**Loss of ADAD2 results in altered abundance of two key DNA damage response proteins.** (A) Western blot of MDC1 and BRCA1 in wild-type and *Adad2^M/M^* 21 dpp testis lysate shows reciprocal impacts on protein abundance in *Adad2* mutants. GAPDH and ADAD2 blots shown as loading and genetic controls (three samples/genotype). The ADAD2 genotype control and GAPDH loading control blots are also shown for the same protein sample panel in Fig. 1D, Fig. S5B,D and Fig. S6D. (B) Immunofluorescence of BRCA1 in adult wild-type and *Adad2^M/M^* testis sections by stage (indicated by Roman numerals) showing increase of XY-body-associated BRCA1 in mutant mid- to late-stage (VII and IX) spermatocytes relative to wild type. Overlays of BRCA1 (green), SYCP3 (red) and DAPI (blue) are shown. Single channel is BRCA1 alone. Inset location is indicated by a square. (C) Immunofluorescence of MDC1 in adult wild-type and *Adad2^M/M^* testis sections by stage showing loss of MDC1 in late meiotic spermatocytes (stages IX and XII). Overlays of MDC1 (green), SYCP3 (red) and DAPI (blue) are shown. Single channel is MDC1 alone. Inset location is indicated by a square. Asterisks indicate metaphase spermatocytes, which are indicative of stage XII.

Combined analysis of total abundance and ribosome association (Fig. 2D) showed cell-cycle and DDR transcripts had a general trend of decreased abundance and increased ribosome association in *Adad2* mutants. Although moderate decreases in abundance may lead to slight reduction in protein production, it is feasible this could be overcome by increased ribosome association. Thus, to test how ribosome association influenced protein abundance in the *Adad2* mutant, we examined two biologically relevant transcripts: one with only slightly increased expression in the *Adad2* mutant (*Brca1*) and another with increased ribosome association in conjunction with moderately reduced transcript abundance (*Mdc1*). Both influence the DNA damage response (Campbell et al., 2010; Lou et al., 2003; Stewart et al., 2003) and meiotic germ cell heterochromatin state, in particular XY-body formation and MSCI (Becherel et al., 2013; Ichijima et al., 2011; Royo et al., 2013), making them potential candidates for mediating heterochromatin defects in *Adad2* mutants. Western blot analysis in 21 dpp wild-type and mutant testes showed a reciprocal pattern of protein abundance, with MDC1 greatly reduced and BRCA1 dramatically increased (Fig. 3A). Although BRCA1 displayed the anticipated response to increased expression, the reduction of MDC1 was unexpected. Thus, we selected several additional transcripts with abundance and ribosome association profiles similar to *Mdc1* and examined their protein abundances (Fig. S5A-C). In all cases, increased ribosome association in conjunction with moderately decreased transcript resulted in dramatically reduced protein abundance to a degree that could not be attributed to transcript abundance decreases.

Increased ribosome association is assumed to be indicative of increased protein production. However, increased ribosome occupancy can lead to reduced protein production and subsequent transcript degradation in cases of either ribosome stalling or reduced translation elongation (Brandman et al., 2012; D'Orazio et al., 2019). Transcript abundance and ribosome association of ribosome stress response transcripts were not substantially altered in *Adad2* mutants, indicating no widespread ribosome stalling. As such, we focused on regulators of translation elongation. Translation elongation requires both the eEF1A complex, which delivers an amino acid charged tRNA to the ribosome, and the eEF1B complex, which serves to activate eEF1A (Sasikumar et al., 2012). Our previous analysis showed eEF1G, a structural component of the eEF1B complex, had reduced protein abundance in *Adad2* mutants (Fig. S5B). Thus, we quantified the abundance of eEF1D, another member of the eEF1B complex, to determine whether eEF1B complex functionality may be reduced in *Adad2* mutants (Fig. S5D). This analysis demonstrated reduction of total eEF1D in both 21 dpp and adult testis, suggesting reduction of the eEF1B complex in *Adad2* mutants. This, together with our analyses of the *Mdc1* transcript and protein, implicate ADAD2 as a regulator of translation elongation in meiotic germ cells and suggest abnormal translation elongation may underpin the *Adad2* phenotype.

### ADAD2 loss leads to cell-specific protein abundance changes in target transcripts

Together, the above results demonstrate that ADAD2 differentially influences the abundance of two targets (BRCA1 and MDC1) that are important for heterochromatin remodeling in meiotic male germ cells, and identify BRCA1 and/or MDC1 as potential mediators of the *Adad2* mutant phenotype. Given both are localized to and closely associated with the formation of the XY-body (Broering et al., 2014; Ichijima et al., 2011; Kogo et al., 2012), we examined whether loss of ADAD2 and the subsequent changes in protein abundance altered their localization (Fig. 3B,C). For both, we observed normal XY-body localization and intensity in early pachytene spermatocytes. However, although BRCA1 showed increased XY-body association in both mid- and late-stage *Adad2^M/M^* spermatocytes, which normally express high levels of granule-localized ADAD2 (Chukrallah et al., 2020), a dramatic reduction of MDC1 was observed exclusively in late-stage spermatocytes. These observations demonstrate that ADAD2 is required to maintain normal BRCA1 and MDC1 protein levels, particularly in late-stage spermatocytes, which influences their relative concentration in the XY-body.

### ADAD2 loss leads to accumulation of γH2AX on autosomes

**Fig. 4.**
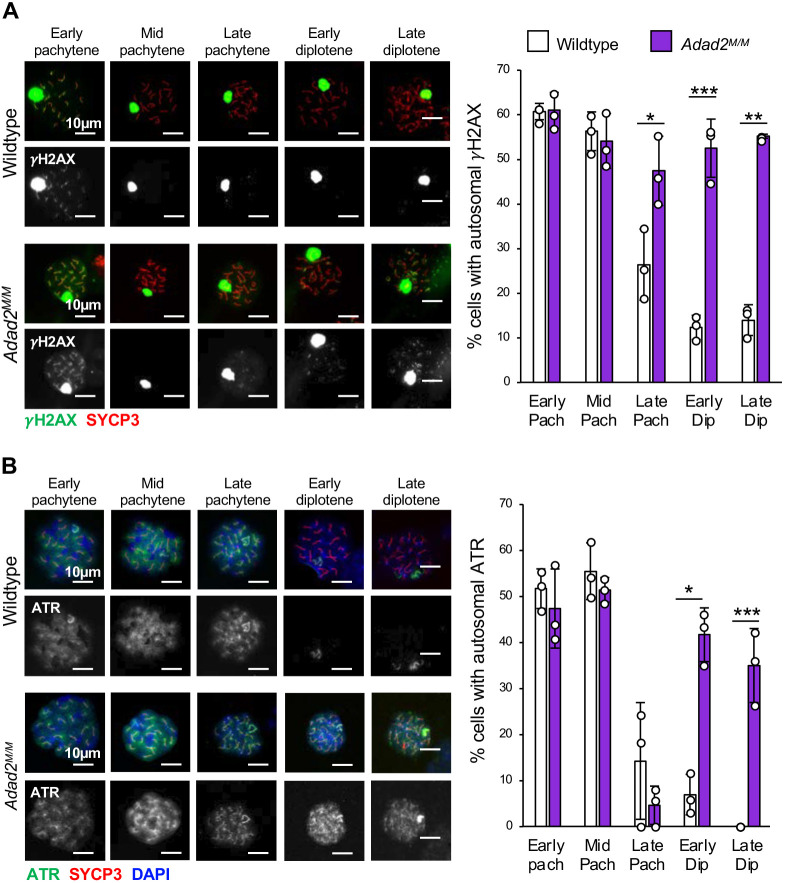
**Loss of ADAD2 results in abnormal autosomal γH2AX and ATR late in meiosis.** (A) Quantification of autosomal γH2AX signal by spermatocyte stage in 30 dpp wild-type and *Adad2^M/M^ *germ cells (H2AX, green; SYCP3, red) showing mutant-specific increase of cells with autosomal γH2AX in late pachytene through diplotene. (B) Quantification of autosomal ATR signal by spermatocyte stage in 30 dpp wild-type and *Adad2^M/M^ *germ cells (three samples per genotype; ATR, green; SYCP3, red; DAPI, blue) showing increased frequency of autosomal ATR in mutant spermatocytes late in meiosis. Images are representative of localization pattern observed in wild type versus mutant. Data are mean±s.d. Dots represent frequencies within individuals. Significance was calculated using an unpaired, two-tailed Student‘s *t-*test (*P<0.05, **P<0.001, ***P<0.0001).

BRCA1 and MDC1 both function in the XY-body to establish a sex chromosome-wide γH2AX domain (Broering et al., 2014; Ichijima et al., 2011). To assess the impact of elevated BRCA1 in conjunction with reduced MDC1, we first examined total γH2AX in 21 dpp whole testes (Fig. S6A), which showed an overall increase. Next, we examined the localization of γH2AX in wild-type and *Adad2* spermatocytes, and found that *Adad2^M/M^* spermatocytes had qualitatively normal levels of γH2AX within the XY-body throughout meiosis, suggesting increased BRCA1 did not result in an expansion of the γH2AX domain. However, a large fraction (50 to 60%) of late pachytene and diplotene *Adad2^M/M^* spermatocytes showed γH2AX along the axes of the autosomes, similar to what is observed in early and mid-pachytene spermatocytes of both genotypes (Fig. 4A). In order to determine whether this aberrant autosomal localization was a result of persistent DNA damage, we quantified RPA2 foci, which mark sites of DNA damage for recombination (Ichijima et al., 2011; Raderschall et al., 1999), in wild-type and *Adad2^M/M^* spermatocytes, and found no significant difference (Fig. S6B,C) demonstrating normal DNA damage repair kinetics in mutant spermatocytes and suggesting the aberrant γH2AX signal is not a result of persistent or recurring DNA damage. Previous work has suggested that some DNA double strand breaks may not be marked by RPA (Lezaja et al., 2021); thus, as a secondary assessment, we examined relative levels of the 9-1-1 DNA damage recognition complex as indicated by RAD9 (Parrilla-Castellar et al., 2004) and activation of NHEJ marked by Ku80 (Fell and Schild-Poulter, 2015; Ma et al., 2018) (Fig. S6D). In both cases, no induction was observed in mutant testes, suggesting normal repair of DSBs in *Adad2^M/M^* spermatocytes.

ATR is initially recruited to the XY-body by BRCA1 early in meiosis, where it phosphorylates H2AX to form γH2AX (Turner et al., 2004). γH2AX, in turn, is bound by MDC1 (Stewart et al., 2003) (Ichijima et al., 2011), which then tethers ATR to the sex chromosomes and facilitates the spread of γH2AX throughout the remainder of the sex chromosomes (Alavattam et al., 2016; Ichijima et al., 2011). To determine whether the abnormal autosomal γH2AX in *Adad2* mutant spermatocytes was a function of mis-localized ATR, we quantified the frequency of *Adad2^M/M^* spermatocytes with autosomal ATR. Although this analysis demonstrated normal axial XY staining of ATR in both wild-type and mutant spermatocytes, it also demonstrated that a sizable number (40 to 50%) of diplotene *Adad2^M/M^* spermatocytes contained ATR along the autosomal axes, a pattern only very rarely observed in the wild type (Fig. 4B). This indicates autosomal γH2AX was likely a function of ATR localization in the diplotene population. Given normal XY-body ATR and γH2AX, we conclude increased BRCA1 had minimal impact on their behavior. In contrast, spreading of ATR to the autosomes implies that loss of MDC1 leads to distinct failure of XY-body γH2AX and ATR maintenance, and suggests that the primary *Adad2* phenotype may be driven in part by MDC1 loss.

### *Adad2* mutant meiotic germ cells display characteristics that are indicative of MDC1 loss

**Fig. 5.**
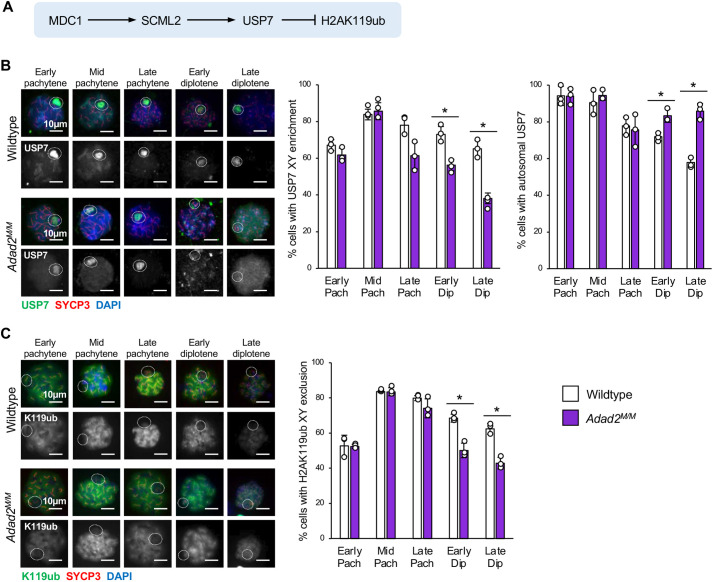
**The MDC1-regulated SCML2 network shifts from the XY-body to the autosome in late-pachytene mutant spermatocytes.** (A) MDC1-mediated regulation of USP7 and H2AK119ub via SCML2. (B) Immunocytochemistry of USP7 and localization pattern quantification by stage in 30 dpp wild-type and *Adad2^M/M^* spermatocyte (three samples per genotype; USP7, green; SYCP3, red; DAPI, blue) showing decreased frequency of XY enrichment and increased frequency of autosomal USP7 signal. Circles indicate the region of the X and Y chromosomes, defined by morphological parameters. (C) Immunocytochemistry of H2AK119ub and localization pattern quantification by stage in 30 dpp wild-type and *Adad2^M/M^* spermatocyte spreads (three samples per genotype; H2AK119ub, green; SYCP3, red; DAPI, blue) showing reduced frequency of H2AK119Ub XY-body exclusion in mutant late meiotic spermatocytes. Images are representative of localization pattern observed in wild type versus mutant. Dashed circles indicate the region containing the X and Y chromosome. Data are mean±s.d. Dots represent frequencies within individuals. Significance was calculated using an unpaired, two-tailed Student‘s *t-*test. *P<0.05.

Although MDC1 is not the only ADAD2-impacted protein, it is a key modulator of epigenetic reprogramming in meiotic male germ cells. Thus, we wondered whether *Adad2* mutant spermatocytes displayed molecular phenotypes indicative of MDC1 loss. We first asked whether well characterized downstream targets of MDC1 were altered in *Adad2* mutant spermatocytes, with an emphasis on late pachytene spermatocytes. In this cell population, SCML2 localizes to the XY-body in an MDC1-dependent manner where it recruits the deubiquitylating enzyme USP7. As a result, in wild-type late pachytene spermatocytes, histone 2A lysine 119 ubiquitylation (H2AK119Ub, referred to here as K119Ub) is partially excluded from the XY-body (Fig. 5A) (Hasegawa et al., 2015; Luo et al., 2015). Hence, mutation of *Scml2* or *Mdc1* results in XY-body accumulation or inclusion of K119Ub, respectively (Adams et al., 2018; Luo et al., 2015). Given the reduction of MDC1 in *Adad2* mutant spermatocytes, we assessed the localization of SCML2 activity in pachytene and diplotene spermatocytes by first examining the localization of USP7 (Fig. 5B). In wild-type spermatocytes the frequency of cells with USP7 XY-body enrichment increased during mid-pachytene and remained high through diplotene, concurrent with a reduction in cells with strong autosome USP7. In contrast, although the majority of mutant spermatocytes established the correct USP7 XY-body enrichment in early and mid-pachytene spermatocytes, the percentage of late pachytene and diplotene spermatocytes with XY-body USP7 decreased significantly in *Adad2* mutants. This was concurrent with an increase of cells with autosomal USP7 and suggests there is a failure to properly localize or maintain the localization of USP7 late in meiosis. We next asked whether this USP7 shift resulted in abnormal deposition of the epigenetic mark K119Ub (Fig. 5C). As expected, the frequency of cells with K119Ub XY-body exclusion was similar between wild type and mutant in early and mid-pachytene spermatocytes but was significantly reduced in mutant late pachytene and diplotene spermatocytes (Fig. 5C). Together, these observations demonstrate a dramatic redistribution of one MDC1-regulated pathway, including the epigenetic mark resulting from it, in *Adad2* mutant spermatocytes at the time of MDC1 reduction. This suggests that adequate MDC1 levels may be necessary to maintain proper localization of downstream networks once established.

In spite of the redistribution of USP7 in the mutant spermatocytes, it remained a possibility that the observed changes resulted from alterations in the expression or ribosome association of known epigenetic mark regulators. To determine whether this was the case, we examined key elements of each pathway using our RNA and RiboTag-sequence analyses (Fig. S5E). Of these, no significant changes in abundance or ribosome association were observed in *Adad2^M/M^* samples, indicating altered epigenetic marks cannot be attributed exclusively to expression or ribosome association changes.

### MSCI is moderately defective in *Adad2* mutants

In early pachytene spermatocytes, MDC1 mediates silencing of sex chromosome expression (MSCI) via deposition or exclusion of specific epigenetic marks specifically on the sex chromosomes (Abe et al., 2020; Ichijima et al., 2011). Given the observed changes to the sex chromosome epigenetic composition, we examined MSCI in *Adad2* mutants by comparing differential expression (DE) across the autosome and sex chromosomes in 21 dpp wild-type and mutant testes (Fig. S7A,B). These analyses demonstrated the X-chromosome was not overrepresented in the DE gene list nor was X-chromosome gene expression significantly increased as a whole. This is in contrast to other models lacking MSCI in which many X-chromosome genes are dramatically upregulated (Turner, 2007). However, in spite of what appeared to be qualitatively normal MSCI in mutant spermatocytes, X-chromosome DE genes were much more likely to be upregulated when compared with autosomal DE genes (Fig. S7C). Although the direct influence of MDC1 on MSCI in late pachytene spermatocytes cannot be assessed due to the *Mdc1* mutant phenotype, mutation of the MDC1 target *Scml2* does result in abnormal MSCI late in meiosis (Luo et al., 2015). Thus, we compared X-chromosome genes upregulated in *Adad2* mutants with those in *Scml2* mutants and found an appreciable overlap (Fig. S7D). Together, these findings suggest MSCI is established normally in *Adad2* mutants but may be maintained improperly, presumably due to insufficient MDC1 protein levels leading to abnormal SCML2 function.

### Deposition of the activating mark H3K4me2 is abnormally regulated in *Adad2* mutants

**Fig. 6.**
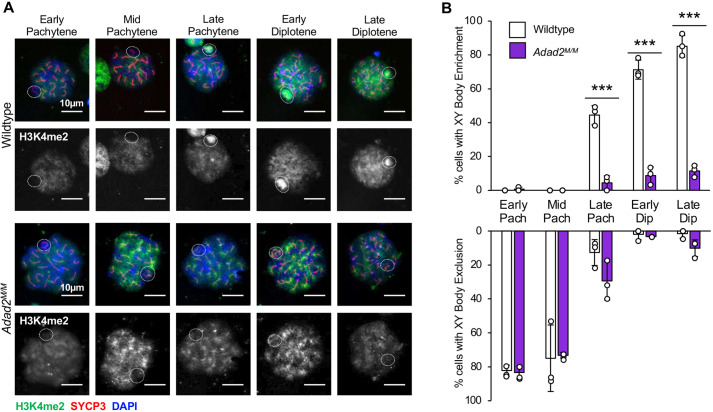
**Mutant spermatocytes fail to localize H3K4me2 to the XY-body late in meiosis.** (A) H3K4me2 immunocytochemistry (H3K4me2, green; SYCP3, red; DAPI, blue; dashed circles indicate regions containing the X and Y chromosome). (B) Quantification of localization pattern in 30 dpp wild-type and *Adad2^M/M^* spermatocytes (biological triplicate per genotype) showing almost complete lack of XY-body accumulation in mutant spermatocytes late in meiosis. Images are representative of the localization pattern observed in wild type versus mutant. Data are mean±s.d. Dots represent frequencies within individuals. Significance is calculated using an unpaired, two-tailed Student‘s *t-*test. ***P<0.0001.

Although the vast majority of differentially expressed X-chromosome genes in the *Adad2* mutant showed increased abundance, a select number demonstrated a decrease. Detailed analysis of these revealed that half (Fig. S7E) belong to the relatively small number of X chromosome-encoded transcripts that undergo activation after the completion of meiosis (Ernst et al., 2019). Reduced expression of these transcripts suggested there may be defects in post-meiotic gene activation of the X-chromosome, which is driven in part by deposition of the activating mark H3K4me2 (Adams et al., 2018). Supporting this notion, our initial heterochromatin characterization (Fig. 1D) demonstrated reduced H3K4me2 in mutant whole testis lysate and spermatocytes. To further explore this, we characterized H3K4me2 XY-body deposition during meiosis, which is dependent on the MDC1-interacting protein RNF8 (Adams et al., 2018). Early in meiosis H3K4me2 is excluded from the XY-body (De Vries et al., 2012; Federici et al., 2015), a finding recapitulated in early and mid-pachytene wild-type and mutant spermatocytes. However, although the frequency of cells with XY-body H3K4me2 enrichment increased dramatically in wild-type spermatocytes from late pachytene to late diplotene, virtually no mutant spermatocytes accumulated XY-body H3K4me2 (Fig. 6). As for other epigenetic marks, we found no significant changes in gene expression or ribosome association for H3K4 methylation genes (Fig. S5E). Together, these analyses suggest ADAD2 indirectly influences H3K4 methylation via its influence on MDC1, in agreement with the reported role of MDC1 in enhancing H3K4me2 in the XY-body. Furthermore, they confirm that the majority of *Adad2* mutant spermatocytes display molecular phenotypes indicative of MDC1 loss in late pachytene spermatocytes.

### *Adad2* mutant spermatids have abnormalities in post-meiotic chromatin and chromatin remodeling

The heterochromatin of post-meiotic spermatids is divided into the chromocenter and a second highly heterochromatic region, termed post-meiotic sex chromatin (PMSC), that contains the condensed X- or Y-chromosomes (Moretti et al., 2016). Given dramatic alterations in meiotic chromatin of *Adad2* mutants and the previously observed abnormalities in chromocenter morphology, we examined epigenetic marks in the heterochromatin of *Adad2* mutant round spermatids.

**Fig. 7.**
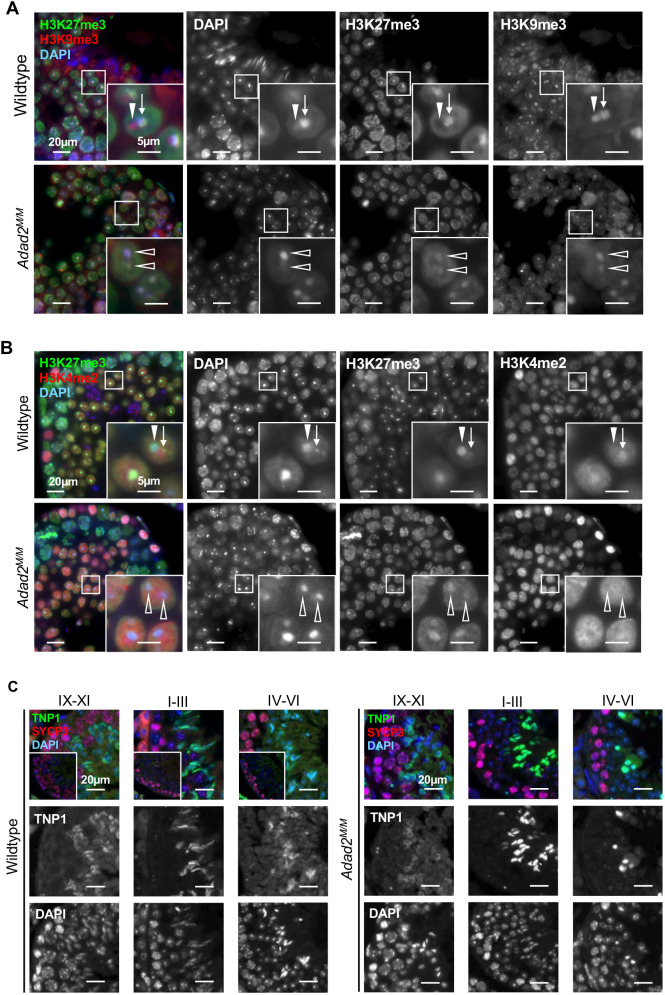
***Adad2* mutant spermatids do not form PMSC and fail to undergo the histone to protamine transition.** (A) Immunofluorescence of H3K27me3 and H3K9me3 in adult wild-type and *Adad2^M/M^ *round spermatids showing mutant round spermatids lack an H3K9me3-only chromatin domain (H3K27me3, green; H3K9me3, red; DAPI, blue). (B) Immunofluorescence of H3K27me3 and H3K4me2 in adult wild-type and *Adad2^M/M^ *round spermatids, demonstrating lack of H3K4me2 enrichment or exclusion in mutant round spermatids (H3K27me3, green; H3K4me2, red; DAPI, blue). In A and B, square indicates the area shown in the inset; arrowheads indicate the PMSC; arrows indicate the chromocenter; open arrowheads indicate the chromocenter-like foci. (C) Immunofluorescence of TNP1 (green) and SYCP3 (red). DAPI is in blue. Insets show stage-matched no anti-TNP1 controls in adult wild-type and mutant testis sections by stage. TNP1 is imported into the nucleus starting in stage XI in wild-type spermatids. It properly associates with the DAPI-dense regions of elongating spermatids by stage I and signal begins to dissipate by stage IV. These events are delayed or abnormal in mutant elongating spermatids.

Each round spermatid heterochromatin region is marked by distinct combinations of histone modifications, with the chromocenter containing both H3K9me3 and H3K27me3, while PMSC is strongly H3K9me3 positive but depleted of H3K27me3 (Ichijima et al., 2012; Turner et al., 2006). Using co-immunofluorescence of H3K9me3 and H3K27me3 in *Adad2^M/M^* round spermatids (Fig. 7A), we observed the fragmented chromocenters of *Adad2^M/M^* round spermatids were marked by both H3K9me3 and H3K27me3, similar to single chromocenters in the wild type. Furthermore, mutant round spermatids did not display either the weakly stained DAPI or H3K9me3-only regions that are indicative of PMSC, suggesting *Adad2^M/M^* spermatocytes lack a distinct PMSC domain. To confirm, we performed co-immunofluorescence of H3K4me2 and H3K27me3 in *Adad2^M/M^* round spermatids (Fig. 7B), with the expectation that regions of PMSC should be strongly H3K4me2 enriched and chromocenters H3K4me2 depleted (Tatehana et al., 2020). This pattern was observed in wild-type round spermatids; however, in the *Adad2* mutant spermatids, H3K4me2 did not localize to any of the chromocenter-like structures nor were other regions of distinct H3K4me2 enrichment observed, demonstrating *Adad2* mutant spermatids lack distinguishable PMSC.

Our earlier TUNEL assays demonstrated a significant increase in round spermatid apoptosis and a general reduction of round spermatids. In spite of this and a lack of epididymal sperm (Chukrallah et al., 2020), occasional morphologically abnormal elongating spermatids are observed in *Adad2* mutant testes. These cells provided an opportunity to examine post-meiotic chromatin remodeling in conditions of abnormal meiotic chromatin. To do so, we examined the localization of the histone replacing protein transition protein 1 (TNP1) (Fig. 7C). In wild-type spermatids, the process of elongation accompanies dramatic reorganization of chromosome centromeres, marked by PCH, along the axis of elongation (Meyer-Ficca et al., 1998). Centromere elongation can be visualized by the DNA staining dye DAPI as spermatids mature. In wild-type elongating spermatids, the single chromocenter (region of intense DAPI staining) gives rise to an elongated region, the formation of which coincides with nuclear import of TNP1. *Adad2* mutant spermatids fail to undergo this chromocenter elongation, even those cells containing a single chromocenter. Furthermore, wild-type spermatids generate TNP1 and import it into the nucleus, where it roughly colocalizes with regions of DAPI in a stage-dependent manner. However, in *Adad2* mutant spermatids, TNP1 nuclear import is delayed and, once imported, TNP1 shows poor colocalization with DAPI staining. Together, these observations suggest that the aberrations in epigenetic marks, and subsequent heterochromatin structures, of mutant meiotic germ cells directly impede their ability to undergo the histone to protamine transition and the concurrent morphological elongation necessary to generate mature sperm.

## DISCUSSION

**Fig. 8.**
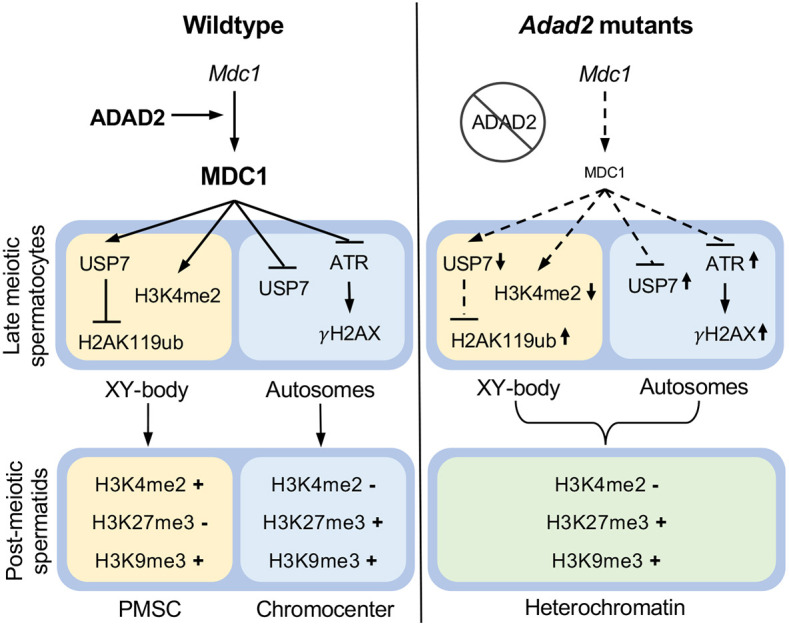
**Summary of ADAD2 action and drivers of the *Adad2* mutant phenotype.** In wild-type germ cells, ADAD2 facilitates translation of *Mdc1* late in meiosis, thus ensuring the correct epigenetic state of the XY body and autosomes, and resulting in two unique heterochromatin domains (PMSC and the chromocenter) in the subsequent post-meiotic germ cells. In the absence of ADAD2, MDC1 is low late in meiosis, leading to abnormal XY-body and autosome epigenetic signatures in late meiotic spermatocytes. In mutant post-meiotic spermatids, improperly marked chromatin domains result in the loss of distinct chromatin compartments.

Heterochromatin remodeling is a hallmark of germ cell differentiation. Herein we show loss of ADAD2, an RNA-binding protein observed exclusively in mid- to late pachytene spermatocytes, leads to abnormal meiotic and post-meiotic heterochromatin in both autosomes and sex chromosomes. This outcome is likely driven in part by reduced translation of *Mdc1* (Fig. 8) late in meiosis. MDC1 is expressed throughout male germ cell meiosis and has previously been associated with establishment of the unique XY-body epigenetic state early in pachynema (Broering et al., 2014; Ichijima et al., 2011), though its role later in meiosis has been enigmatic. In *Adad2* mutants, MDC1-dependent XY-body epigenetic marks (Adams et al., 2018; Alavattam et al., 2016; Sin et al., 2012) are established properly but fail to be maintained through late meiosis. Additionally, γH2AX and ATR are aberrantly observed on autosomal axes late in meiosis in mutant spermatocytes. Although *Adad2* mutant spermatocytes complete meiosis, autosomal and sex chromosome heterochromatin is severely perturbed in post-meiotic spermatids, resulting in spermatid apoptosis and failed chromatin compaction. These results explain the loss of post-meiotic germ cells in *Adad2* mutants and additionally suggest that *Adad2* mutants may be a unique model with which to investigate the function of MDC1 late in meiosis.

Although the underlying molecular driver of the *Adad2* phenotype likely arises during mid- to late pachytene, at the physiological level, ADAD2 loss leads to significant reduction in post-meiotic germ cells (Snyder et al., 2020). We propose this cell loss occurs over two phases: the first at or just after the transition from spermatocyte to spermatid and the second during the round spermatid to elongating spermatid transition. These results demonstrate that round spermatid development per se is not heavily reliant on proper heterochromatin distribution, whereas cell type transitions involving chromatin rearrangements, such as completion of meiosis and the histone to protamine transition are, further supporting the notion that abnormal heterochromatin is the primary driver of the *Adad2* mutant phenotype.

At the molecular level, the loss of MDC1 in *Adad2* mutants appears to be counterintuitive as the *Mdc1* transcript shows dramatically increased ribosome association, normally associated with increased translation. However, several other transcripts also demonstrate this unusual signature, wherein overall transcript abundance is minimally decreased, ribosome association is increased and protein abundance is dramatically reduced. Evidence from other systems has shown this phenomenon is indicative of abnormal translation elongation dynamics (Simms et al., 2017; Yan and Zaher, 2021). Translation elongation is increasingly being recognized as an important aspect of translation control that can have a profound influence on cell physiology. Elongation rates are exceptionally dynamic during meiosis in yeast (Sabi and Tuller, 2019) and profoundly influence protein production during early embryogenesis (Richter and Coller, 2015). Furthermore, evidence from mitotic models has demonstrated that elongation can be regulated via post-translational regulation of the core elongation complex (Sivan et al., 2011). In spite of this, translation elongation is almost entirely unstudied in the male germ cell. The dramatic reduction of two members of the key elongation complex eEF1B in *Adad2* mutants makes it tempting to postulate that ADAD2 may be required for normal translation elongation in meiotic germ cells. The exact mechanism by which ADAD2 influences the eEF1B complex is, as yet, unknown. Ongoing and future efforts are aimed at answering this exciting question.

In this report, we focus on the impact of ADAD2 on DNA damage response proteins. However, it is important to note that ADAD2 likely regulates other important aspects of germ cell biology. Of particular interest is our observation that transcripts encoding translation and ribosome assembly factors or RNA processing proteins have altered ribosome association in *Adad2* mutants. Given the tight reliance of the germ cell on post-transcriptional regulation (Braun et al., 1989; Kleene et al., 1984), it seems likely these mechanisms may be driving additional biology not explained by abnormal DDR.

In spite of other pathways with abnormal ribosome association in *Adad2* mutants, deregulation of DDR-mediated events, in particular MDC1, explains a large part of the *Adad2* phenotype and provides novel insight into the function of MDC1 late in meiosis. First, the opposing impact of ADAD2 loss on BRCA1 and MDC1 provides a platform to dissect the relative contributions of both, late in meiosis. Our findings support the notion that although BRCA1 acts as a nucleating factor in the XY-body for MDC1 recognition of γH2AX early in meiosis (Broering et al., 2014; Scully et al., 1997; Turner et al., 2004), thereafter BRCA1 alone is not sufficient to maintain sequestration of ATR to the XY-body, demonstrating a key role of MDC1 in this process. Second, two MDC1-influenced epigenetic marks, H2K119Ub and H3K4me2 (Adams et al., 2018; Maezawa et al., 2018; Sin et al., 2012), have abnormal abundance and/or shifts from their normal autosomal or sex chromatin domains in *Adad2* mutant cells. Additionally, lack of detectable PMSC in *Adad2* mutants demonstrates failed H3K27me3 exclusion from the X and Y during meiosis, which is normally prevented by MDC1 (Ichijima et al., 2011). Together, these findings demonstrate MDC1 is required in mid- to late meiosis to ensure specific epigenetic compartments in the autosomes and sex chromosomes of male germ cells.

Previous work has proposed MDC1 facilitates establishment of the γH2AX domain on the sex chromosomes via tethering of ATR and TOPBP1 (Abe et al., 2020; Ichijima et al., 2011). Redistribution of ATR and γH2AX late in meiosis in *Adad2* mutant spermatocytes demonstrates MDC1 tethering is also required throughout the remainder of meiosis, while increased total γH2AX suggests MDC1 may also suppress ATR activity. This is further supported by the observation of increased H3K9me3 in *Adad2* mutant testes as the ATR/γH2AX network is upstream of SETDB1, which deposits H3K9me3 (Hirota et al., 2018). Future studies will be required to determine how ATR recognizes autosome axes when XY-body MDC1 is low. Whether MDC1 regulates other aspects of DNA damage repair or meiotic progression late in meiosis, as has been demonstrated for early meiosis (Testa et al., 2018), is unknown. Future studies leveraging the *Adad2* mutant may provide valuable insight into this issue.

Meiosis is not the only context in which MDC1 appears to tether components to regions containing γH2AX. During mitosis, DNA repair and DNA damage checkpoints are suppressed in order to ensure chromosome stability (Rieder and Cole, 1998). As such, mitotic cells rely on MDC1 to bind γH2AX at sites of DNA damage and mark them for repair during the next G1 phase (Lee, 2014; Orthwein et al., 2014). Recent work has shown MDC1 recruits and tethers TOPBP1 in mitosis to sites of DNA damage, thus forming a repair poised complex (Leimbacher et al., 2019). Meiotic cells represent another system in which DNA repair is suppressed (Srivastava and Raman, 2007) and, based on this work, also rely on the tethering function of MDC1 throughout cell division. As the composition of the mitotic DNA bound MDC1 complex remains undefined, it seems feasible MDC1 may also mediate epigenetic remodeling in mitosis at sites of DNA damage.

This work demonstrates ADAD2 is required to maintain a proper epigenetic state late in meiosis. Importantly, abnormalities in epigenetic marks are retained in mutant germ cells from late meiosis into post-meiotic spermatids, which show dramatic alterations in both autosome and sex chromosome heterochromatin. Only a handful of mutant models display similar post-meiotic germ cell heterochromatin defects to those observed in *Adad2* mutants (Maezawa et al., 2018; Martianov et al., 2002; Shang et al., 2007). In each, the causative events initiate late in meiosis and lead to substantial post-meiotic germ cell differentiation defects. These and the findings reported here demonstrate that post-meiotic germ cell differentiation is highly reliant on proper heterochromatin remodeling late in meiosis. Furthermore, they suggest meiotic germ cells lack a heterochromatin-dependent checkpoint late in meiosis, identifying mid- to late pachytene as a period in germ cell development that may be especially sensitive to the influence of epigenetic modifiers.

Overall, our findings define the mechanism whereby ADAD2 expression in meiotic germ cells leads to post-meiotic germ cell loss and reveal a heretofore unappreciated role for MDC1 in the maintenance of XY-body epigenetic state late in meiosis, underscoring the importance of MDC1 throughout meiosis and beyond. Last, we observed an unanticipated mode of translation regulation by ADAD2. The subsequent new avenues of study should shed light on an understudied level of translation regulation in meiotic germ cells and its connection to heterochromatin and the cell cycle.

## MATERIALS AND METHODS

### Animal care and model generation

All animal use protocols were approved by the Rutgers University animal care and use committees. Mouse procedures were conducted according to relevant national and international guidelines (AALAC and IACUC). Generation of *Adad2^M/M^* mice was as described by Chukrallah et al. (2020). RiboTag mice (Sanz et al., 2009) were obtained from The Jackson Laboratory and *Adad2-Ribotag* mice were generated as described by Chukrallah et al. (2020)*.* Mice were housed in a sterile, climate-controlled facility on a 12 h light cycle. Mice were fed LabDiet 5058 irradiated rodent chow and had access to food and water *ad libitum.*

### Germ cell separation

Spermatocytes and round spermatids were isolated from pooled testes using an adaptation of the STA-PUT protocol (Miller and Phillips, 1969) as described by Dunleavy et al. (2019). In order to capture each population of interest, testes from 50-70 dpp animals were used. These testes include all stages of meiotic and post-meiotic germ cell development. In brief, testes from 50-70 dpp wild-type and *Adad2^M/M^* mice (*n*=4 per genotype) were pooled. Testes were de-tunicated and incubated in both a collagenase and a trypsin solution at 37°C with gentle rocking to dissociate germ cells. Cells were filtered and then purified on a BSA gradient for 4 h at room temperature. Fractions were collected as described by Dunleavy et al. (2019). Part of each fraction was reserved for immunocytochemical analysis of composition.

Fraction purity was assessed via immunocytochemical analyses of cell types, confirming fraction 3 as spermatocyte enriched (SYPC3+) and fraction 5 as round spermatid enriched [determined by nuclear morphology (Fig. S1B)]. The remainder of the purified fractions was split and underwent either whole-protein extraction with RIPA or histone-specific extraction, as described below.

### Preparation of meiotic spreads and immunocytochemistry

Meiotic spreads were prepared following a modified version of the protocol described by La Salle et al. (2008). Testes from 30 dpp animals were used as they contain the full complement of meiotic cells along with the round spermatids of all developmental stages with limited numbers of elongating or elongated spermatids. In brief, testes from 30 dpp *Adad2^M/M^* and wild-type male mice were collected in 1× PBS and lysed in a hypotonic extraction buffer [30 mM Tris-HCl pH 8.2, 50 mM sucrose, 17 mM sodium citrate, 5 mM EDTA, 2.5 mM DTT and 0.5 mM PMSF] for 15 min at room temperature. Tubules were transferred to and dispersed in a 100 mM sucrose solution. Tubule solution (20 µl) was spread on charged slides coated with 1% PFA solution plus 0.14% Triton X-100 and left in a humid chamber to dry overnight. Slides were washed for 2 min in 0.4% Kodak Photoflo and air-dried at room temperature before either using directly for immunocytochemistry or stored at −20°C for later use.

Prior to staining, slides were blocked for 1 h at room temperature in 1× ADB (45 mM BSA, 1% normal goat serum and 0.2% Triton X-100 in 1× PBS). Primary antibodies were diluted in 1× ADB and incubated overnight in a humid chamber either at 4°C or room temperature. Slides were then rinsed in 1× PBS for 10 min, then twice in 1× ADB for 10 min each. Fluorescent secondary antibodies were diluted in 1× ADB. Slides were incubated in light-protected humid chamber for 2 h at room temperature, then washed in 1× PBS for 10 min three times. Slides were mounted using DAPI Fluoromount-G (SouthernBiotech) and stored at 4°C with light protection. For a detailed breakdown of antibody concentrations and conditions, please see Fig. S8A.

Spermatocyte stages were distinguished by SYCP3 pattern as described previously (Luo et al., 2015). Briefly, X- and Y-chromosome structure was used to distinguish early, mid and late pachytene spermatocytes. Early pachytenes were defined by strong SYCP3 staining with shorter strands and no overtly obvious X- and Y-chromosome complex, mid-pachytenes possessed relatively longer SYCP3 strands and a distinct X- and Y-chromosome, and late pachytenes characterized by distinct, defined bulbs at the ends of the synaptonemal complex. De-synapsis of three or fewer SYCP3 strands indicated early diplotene and greater than three late diplotene. Cells were imaged using a custom-built Zeiss microscope with bright-field and fluorescent capabilities. Each channel was imaged individually through MetaMorph imaging software (Molecular Devices) and color-combined using the built-in color combine tool of the program. Provided images are representative of three or more biological samples. With the exception of RPA2, all quantification was carried out via direct visualization. For all quantifications, three wild-type and three *Adad2^M/M^* samples were used. A minimum of 40 cells per cell type per individual were assessed for staining pattern, with the exception of early diplotene (total of 120 per genotype), of which 30 cells were assessed due to the low frequency. Percent means were calculated at the level of biological replicate and s.d. is across biological replicates. Significance was calculated using an unpaired, two-tailed Student's *t*-test.

### RPA2 foci quantification

Thirty spermatocytes at each stage (early, mid and late pachytene, as well as diplotene) were imaged per sample (30 dpp wild type and *Adad2^M/M^*; *n*=3). For each, an RPA2 image, an SYCP3 image and a DAPI image were taken. For each cell, RPA2 foci were quantified using ImageJ (Abramoff04) and the Analyze Particle function (minimum size=10 pixels; threshold=50). Cell boundary was determined by DAPI signal and stage by SYCP3, as above. The number of discrete foci was recorded and the mean was calculated by spermatocyte stage and genotype. Data were visualized in R.

### Protein isolation and western blotting

Testes were collected from adult (60-70 dpp) and 21 dpp *Adad2^M/M^* and wild-type male mice and flash frozen. Tissue was ground in liquid nitrogen and total protein was extracted by RIPA buffer with protease inhibitors at a ratio of 1 ml buffer to 100 mg tissue. Protein concentration was determined via the DC protein assay (BioRad) as described by Chukrallah et al. (2020). Either 20 µg protein or 10 µg for purified cells was electrophoresed on 10% acrylamide gels per sample and Coomassie Blue was used to verify equal protein loading. Following wet transfer of proteins to a PVDF membrane (BioRad), membranes were blocked and incubated overnight with primary antibody at 4°C. Images were developed with SuperSignal West Pico PLUS Chemiluminescent Substrate (Thermo Scientific) and visualized using an Azure Biosystems C600 imager. For a detailed breakdown of antibody concentrations and conditions, please see Fig. S8A. For each age, a single protein panel of biological replicates (n=3 per genotype for most analyses) was generated. Proteins were quantified, diluted to a concentration of 1 mg/ml and utilized for all western blot analyses. Sample order was held constant across all western blot analyses. For each protein panel, a single genotype control (ADAD2) and loading control (GAPDH) was generated. These controls are shown across multiple figures (Fig. 1D, Fig. 3A, Fig. S5B,D and Fig. S6D) to aid in reader analysis. In the case of eEF1D in 21 dpp testes (Fig. S5D), n=2 per genotype as the first wild-type sample and the last mutant sample were unavailable. The associated ADAD2 and GAPDH control blot images were cropped to remove these samples, and thus they align with the presented eEF1D samples.

Western blot band intensity was quantified using ImageJ as described previously (Janes, 2015). A band intensity value was calculated for each sample in the blot using Analyze Gel tool in ImageJ. As this tool calculates band density with respect to background, all quantifications are of the signal intensity relative to the level of background. The band density of the experimental blot was normalized by taking the ratio of the density of the experimental band to the density of the corresponding GAPDH or Total H3 (histone extraction only) band. Mean values for wild-type and *Adad2^M/M^* sample values were calculated. Standard deviation was calculated, and significant differences determined using an unpaired, two-tailed Student's *t*-test.

### Extraction and western blotting of histones

Testes were collected from adult (60-70 dpp) and 21 dpp *Adad2^M/M^* and wild-type male mice, ground on dry ice and resuspended in Triton extraction buffer (TEB) [0.5% Triton X-100, 2 mM phenylmethylsulfonyl fluoride (PMSF) and 0.02% NaN_3_] at 100 mg tissue/ml buffer. Tissue was lyzed by rotating at 4°C for 10 min, then centrifuged at 2000 ***g*** for 10 min at 4°C. Supernatant was discarded, and the pellet was resuspended in half the starting volume of TEB and centrifuged as above. The pellet was resuspended in an equal volume of 0.2N HCl and acid-extracted overnight at 4°C. The sample was then centrifuged at 2000 ***g*** for 10 min at 4°C and supernatant collected. Protein content was determined using the BioRad DC protein assay, as above, and equal loading confirmed by Coomassie Blue. Either 20 µg histone lysate or 10 µg for purified cells was electrophoresed on 15% acrylamide gels and transferred to PVDF membranes as above. Membranes were washed in 1× PBS with 0.1% Triton and blocked in 5% BSA in 1× PBS with 0.1% Triton. Membranes were visualized and imaged as above. For a detailed breakdown of antibody concentrations and conditions, please see Fig. S8.

### Histological evaluation

Adult and 21 dpp wild-type and *Adad2^M/M^* testes were collected and fixed overnight in Bouin's solution (Sigma Aldrich). Tissue was cleared in deionized H_2_O, dehydrated in increasing concentrations of ethanol, embedded in paraffin wax and cut into 4 μm sections. Slides were deparaffinized and rehydrated before staining with Periodic Acid (Millipore Sigma), Schiff's Reagent (Millipore Sigma) and Meyer's Hematoxylin (Millipore Sigma). Slides were dehydrated in ethanol and mounted with Permount mounting medium (Fisher Scientific).

Histological parameters previously described (Russel et al., 1990) were used to (1) quantify the number of round spermatids per tubule and number of round spermatid-containing tubules per 21 dpp sample; and (2) the number of MII spermatocytes (*n*>100/sample) per tubule and the number of stage XII tubules (*n*=50) per adult sample. For both counts, totals and averages (means) for each genotype were calculated, as well as s.d. An unpaired, two-tailed Student's *t-*test was used to identify significant differences by genotype.

Round spermatid morphology was quantified in DAPI-stained samples (21 dpp wild type and *Adad2^M/M^*, *n*=3). At least 1200 round spermatids were quantified per sample. Spermatids were binned by number of intensely DAPI-staining structures (1, 2 or 3+). Means were calculated by genotype percent of each pattern. Standard deviation was calculated, and significant differences determined using an unpaired, two-tailed Student's *t*-test.

### Immunofluorescence

Testes were dissected from adult mice and fixed overnight in 4% PFA. Tissue was rinsed in PBS and dehydrated in increasing concentrations of ethanol before embedding in paraffin wax. All applications used 4 μm sections. Antigen retrieval was performed by boiling slides in either 14.3 mM citrate solution (pH 5.95) for 7 min, 10 mM glycine (pH 2.5) for 8 min or Tris- EDTA [10 mM Tris-HCl, 1 mM EDTA and 0.05% Tween (pH 9.0)] for 30 min. Slides were mounted using DAPI Fluoromount-G and stored at 4°C with light protection. Slides were visualized on a custom-built microscope (Zeiss) with fluorescent and bright-field capabilities. Provided images are representative of three or more biological samples. Signal intensity was matched across slides by matching background (interstitial) signal intensity Developmental stages were determined according to the parameters set forth by Russel et al. (1990), facilitated by SYCP3 co-staining where possible.

### Tubule staging criteria

Stages of seminiferous tubule sections were determine according to the definitions outlined previously (Russel et al., 1990), using a combination of SYCP3 and DAPI staining, and are further outlined in Fig. S1A. As *Adad2^M/M^* males do not complete spermatogenesis, staging was reliant on cell types present prior to ADAD2 expression, primarily preleptotene, leptotene and zygotene spermatocytes, which allowed for bins of stages to be identified. For tubules, staging was binned as follows: I-III, early pachytene spermatocytes; IV-VI, mid-pachytene spermatocytes; VII-VIII, preleptotene and late pachytene spermatocytes; IX-XI, leptotene and diplotene spermatocytes; XII, zygotene spermatocytes and two or more metaphase, anaphase, telophase or MII spermatocytes.

### TUNEL assay

Adult testes from wild-type and *Adad2^M/M^* mice were fixed in PFA, embedded in paraffin wax and sectioned as above. Slides were deparaffinized with xylenes and rehydrated in decreasing concentrations of ethanol (100%, 95%, 70% and 50%) before incubation at 37°C in Proteinase K (1:500 in 1× PBS). TMR Red In Situ cell death detection kit (Millipore Sigma) was used, as per manufacturer's instructions. Slides were mounted using DAPI Fluoromount-G and stored at 4°C with light protection. TUNEL-positive cells were quantified by direct visualization. Cells determined via DAPI staining to be either spermatocytes or spermatids were quantified, as were the number of these cells with a distinct red TUNEL signal in the triple channel. A minimum of 100 cells per each type were quantified per sample (*n*=3 for wild type and *Adad2^M/M^*). From this, the percentage of TUNEL-positive cells was calculated for each cell type in each sample, then averaged (mean) by genotype. Standard deviation was calculated and significant differences determined using an unpaired, two-tailed Student's *t*-test.

### Ribotag RNA immunoprecipitation

Testes were collected from 21 dpp males heterozygous for *Rpl22-HA* and *Stra8 iCre* that were either *Adad2^M/M^* (*n*=4) or *Adad2^+/+^* (*n*=3). Immunoprecipitation and RNA extraction were carried out as described previously (Chukrallah et al., 2020). In brief, testes were homogenized in lysis buffer and precleared twice using antibody-free beads, and pre-immunoprecipitation (input) fractions were collected. Overnight incubation with an anti-HA antibody (ABCAM) was followed by a 2 h incubation with Protein A Dynabeads (Invitrogen). Beads were washed and RNA was extracted using the miRNeasy mini kit (Qiagen) according to the manufacturer's instructions. Each biological replicate generated a total RNA sample (input) and an immunoprecipitated (IP) RNA sample.

### RNA-sequencing and quality control

Input and IP samples were quantified via Nanodrop and assessed for RNA integrity using an Agilent Bioanalyzer (Agilent RNA 6000 Pico). Sample RNA was sent to Genewiz for commercial sequencing (Total RNA, Illumina HiSeq 4000, paired end, 150 bp reads). Strand-specific libraries were prepped with the Ribo-zero Gold HMR and TruSeq Stranded Total RNA Library Prep Human/Mouse/Rat. Raw reads and processed files for this analysis are available in GEO under accession number GSE190525.

Following sequencing, read quality was assessed using the FastQC software from Babraham Bioinformatics and summarized with MultiQC (V1.9; Ewels et al., 2016). *In silico* rRNA depletion was carried out using BOWTIE2 (V2.4.1; Langmead and Salzberg, 2012). Based on initial quality reports, the first 20 bp and last 30 bp were trimmed (TRIMMOMATIC; Bolger et al., 2014) generating 100 bp, paired-end reads. A final rerun of FastQC and MultiQC indicated improved quality. Principle component analyses to confirm sample ID assignment were carried out using pcaExplorer (Marini and Binder, 2019).

### Data analyses

An expanded testis transcriptome was generated by appending novel testis-specific genes and isoforms (Gamble et al., 2020) to the current Ensembl mouse transcriptome (Mus_musculus.GRCm38.90, mm10). Briefly, single-end strand-specific RNA-seq reads derived from isolated testicular cell types were taken from the SRA database (GEO accession numbers GSE43717, GSE43719 and GSE43721; Soumillon et al., 2013) and aligned to the transcriptome. Reads were aligned to this expanded transcriptome and abundance estimated using RSEM (v 1.3.3) (Li and Dewey, 2011). For input samples, differential expression was calculated by EBSeq (release 3.12) (Seirup et al., 2020) with a cutoff of PPDE≥0.95 and a directional fold change greater than one. Ribosomal association (RA) was determined by calculating the ratio of IP TPM over input TPM. Any transcripts with any input values equaling zero were removed. A Welch's unpaired, two-tailed *t*-test was conducted using R (matrixTests) to identify differential ribosome association (DRA, *P*<0.05). Heat maps were generated using R (base R) as described previously (Snyder et al., 2020) using publicly available datasets. The percentage total expression for both DE and DRA genes by cell type was plotted in terms of abundance. Graphical representations of data as scatter plots (base R) and violin plots (ggplot2) were generated using R. Ontological analyses were performed using the DAVID bioinformatics database (Huang da et al., 2009). Ontological categories were identified from gene lists differentially expressed or differentially ribosome associated in wild-type or *Adad2^M/M^* samples. Clusters were identified using a medium classification stringency and *P* values were confirmed to be statistically significant (*P*<0.05).

## Supplementary Material

10.1242/joces.259196_sup1 Supplementary information
