# Supplementary material for: ADAD2 regulates heterochromatin in meiotic and post-meiotic male germ cells via translation of MDC1
Source: J Cell Sci. 2022 Feb 22;135(4):jcs259196. doi: 10.1242/jcs.259196 (PMC8919335; doi:10.1242/jcs.259196)
Supplement: Supplementary information [file joces-135-259196-s1.pdf]

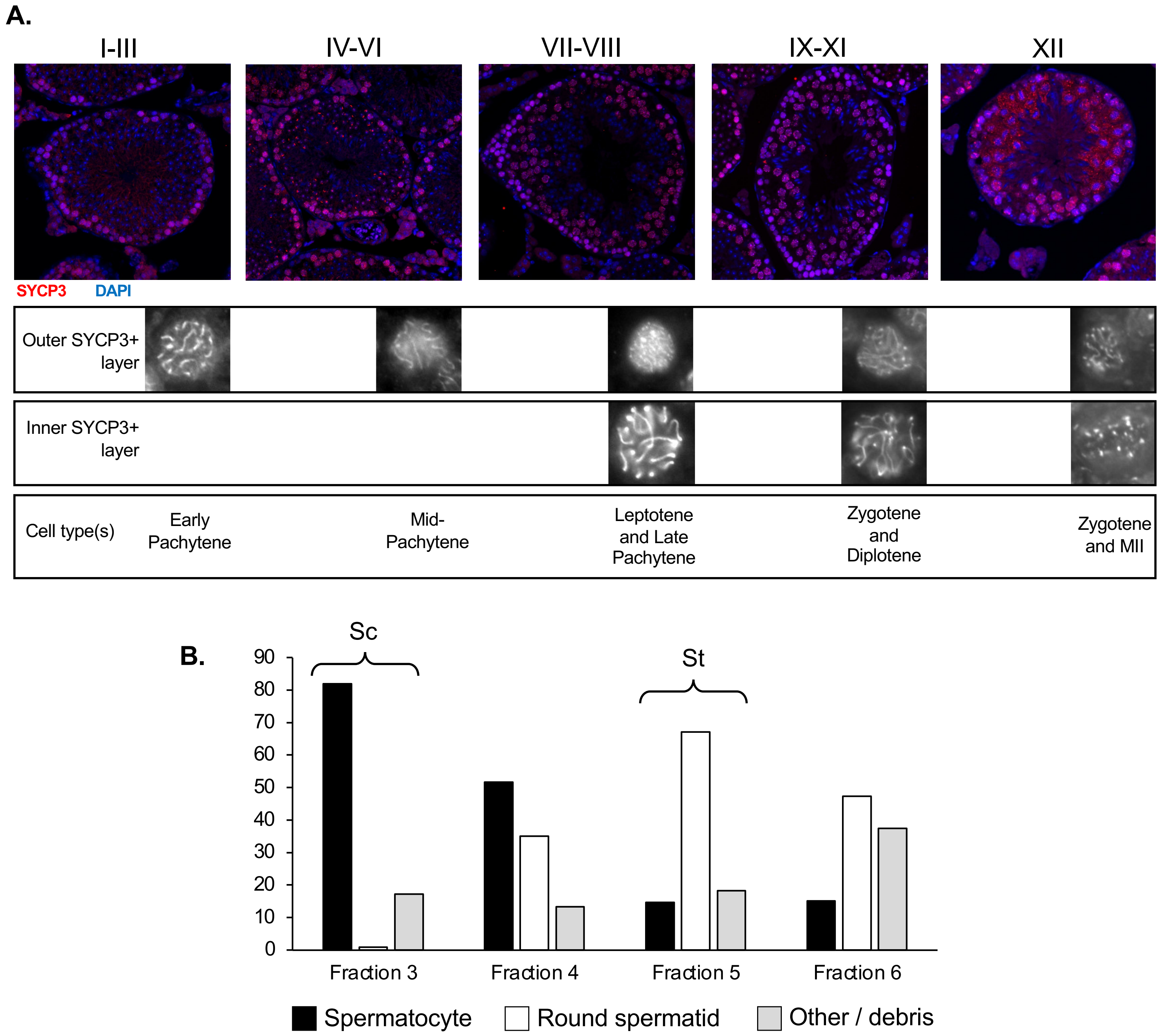

Fig. S1. **A)** Criteria for staging by immunofluorescent SYCP3 staining. Tubules were examined for the number of SYCP3+ cell layers followed by analysis of SYCP3 staining pattern to identify spermatocyte type. **C)** Quantification of cell enrichment by STAPUT demonstrating greater than 70% enrichment in selected fractions (Sc – selected spermatocyte fraction, St – selected round spermatid fraction).

Journal of Cell Science • Supplementary information

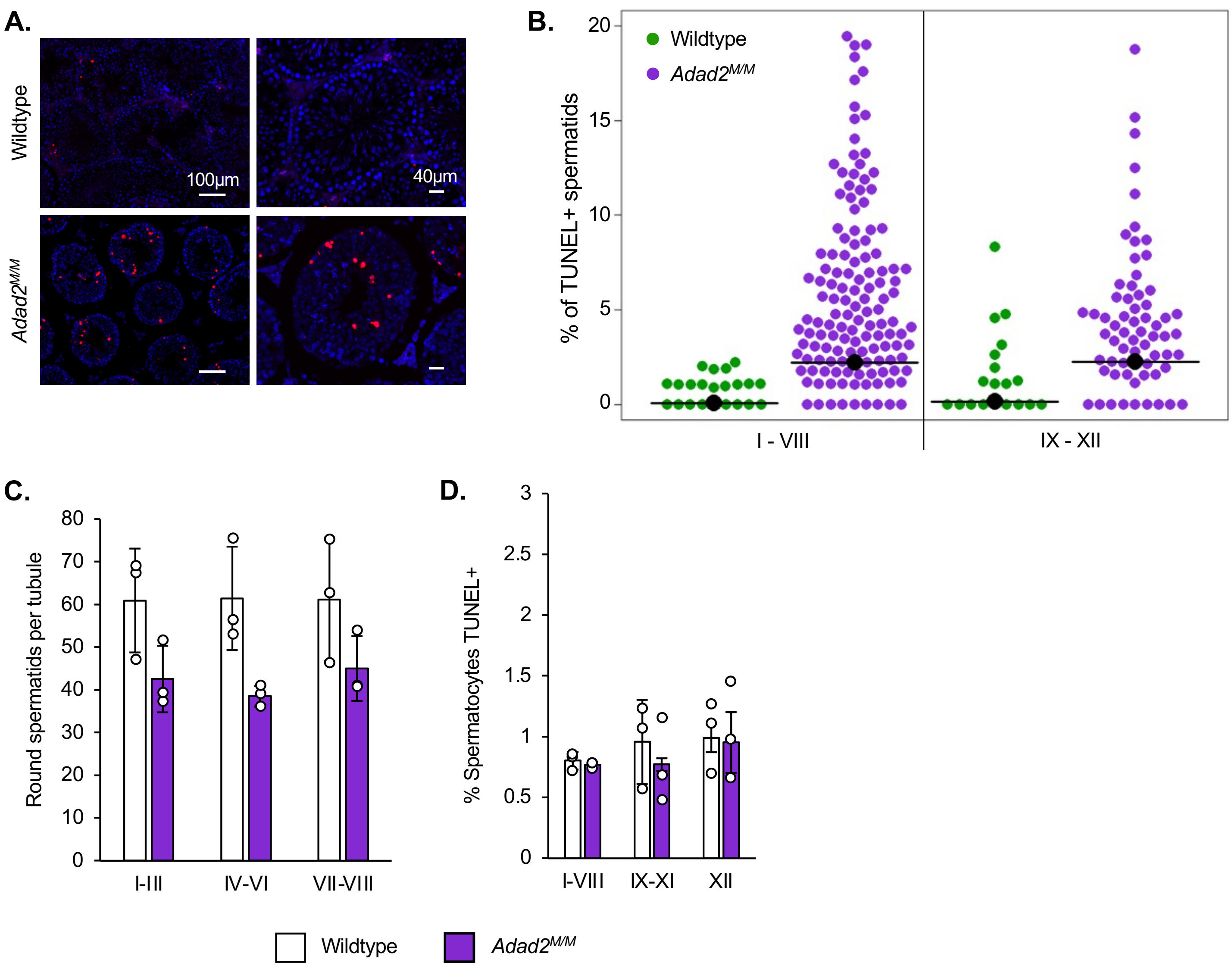

Fig. S2. Apoptosis in *Adad2* mutants is limited to post-meiotic germ cells. **A)** TUNEL staining (red -TUNEL+, blue - DAPI, upper panels – 20x, lower panels – 40x) in wildtype and *Adad2*$^{M/M}$ testis sections. **B)** was used for quantification of apoptotic cells in wildtype and *Adad2*$^{M/M}$ adult testis sections. (n = 3/genotype). Note: the number of tubules containing zero apoptotic cells was artificially reduced to facilitate plotting. 92-94% of wildtype tubules contained zero apoptotic cells, while 55-65% of mutant tubules did, regardless of stage. **C)** Round spermatids per tubule as a function of stage
(assessed by SYCP3 staining) showing a reduced round spermatid population throughout development. Plotted points represent individual samples. **D**) Frequency of TUNEL+ spermatocytes by stage which shows no change relative to wildtype in mutant spermatocytes**.** Plotted points represent individual samples.

Journal of Cell Science • Supplementary information

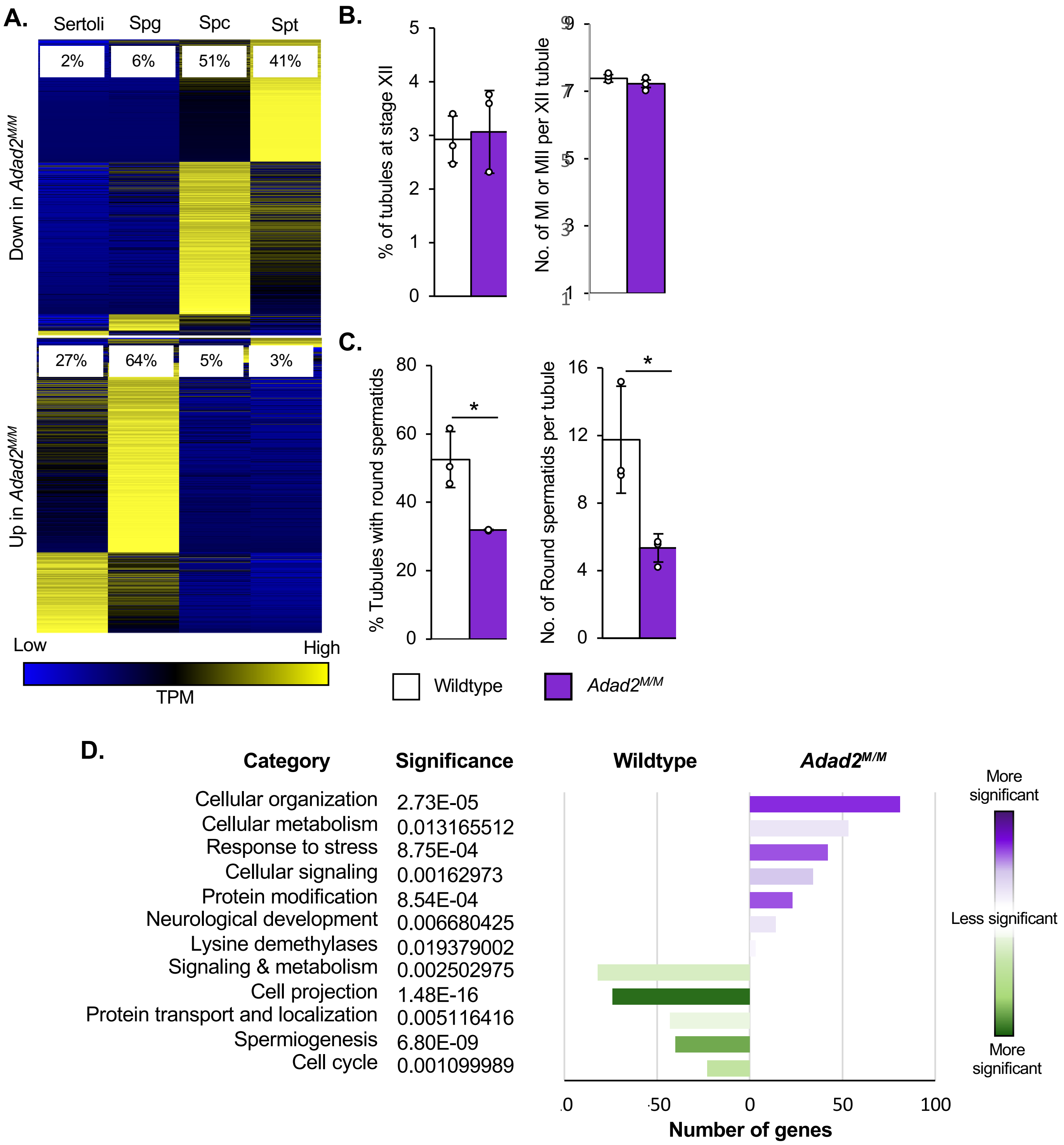

Fig. S3. Reduced expression of spermatocyte transcripts in *Adad2* mutants is not a function of spermatocyte loss. A) Expression of DE genes in isolated wildtype cells (data derived from cell-specific RNA-sequencing datasets (Soumillon et al., 2013)) shows genes down-regulated in *Adad2* mutant testes are generally expressed in wildtype meiotic spermatocytes (Spc) and post-meiotic spermatids (Spt) as opposed to mitotic spermatogonia (Spg) or somatic (Sertoli) cells while DE genes up-regulated in *Adad2* mutant testes are predominantly expressed in spermatogonia. **B)** Quantification of stage XII tubules, which contain late meiotic spermatocytes (left), and the average number of late phase (metaphase, telophase, or anaphase I or MII) spermatocytes (right) in adult wildtype (n = 3) and mutant (n = 3) testis cross sections shows no reduction of late meiotic germ cells in mutant testes. **C)** Round spermatid quantification, expressed as tubules with round spermatids and round spermatid per tubule, in 21dpp wildtype (n = 3) and mutant (n = 3) testis cross sections shows a significant reduction of round spermatids in mutant testes. **D)** Ontological analysis of DE transcripts (up in wildtype - green and up in *Adad2*$^{M/M}$ - purple) shows enrichment of specific classes by genotype. For B and C, plotted points represent means of cells per tubule within biological replicates, error bars represent one standard deviation and significance was calculated using Student's *t*-test with * $p < 0.05$, ** $p < 0.001$, *** $p < 0.0001$.

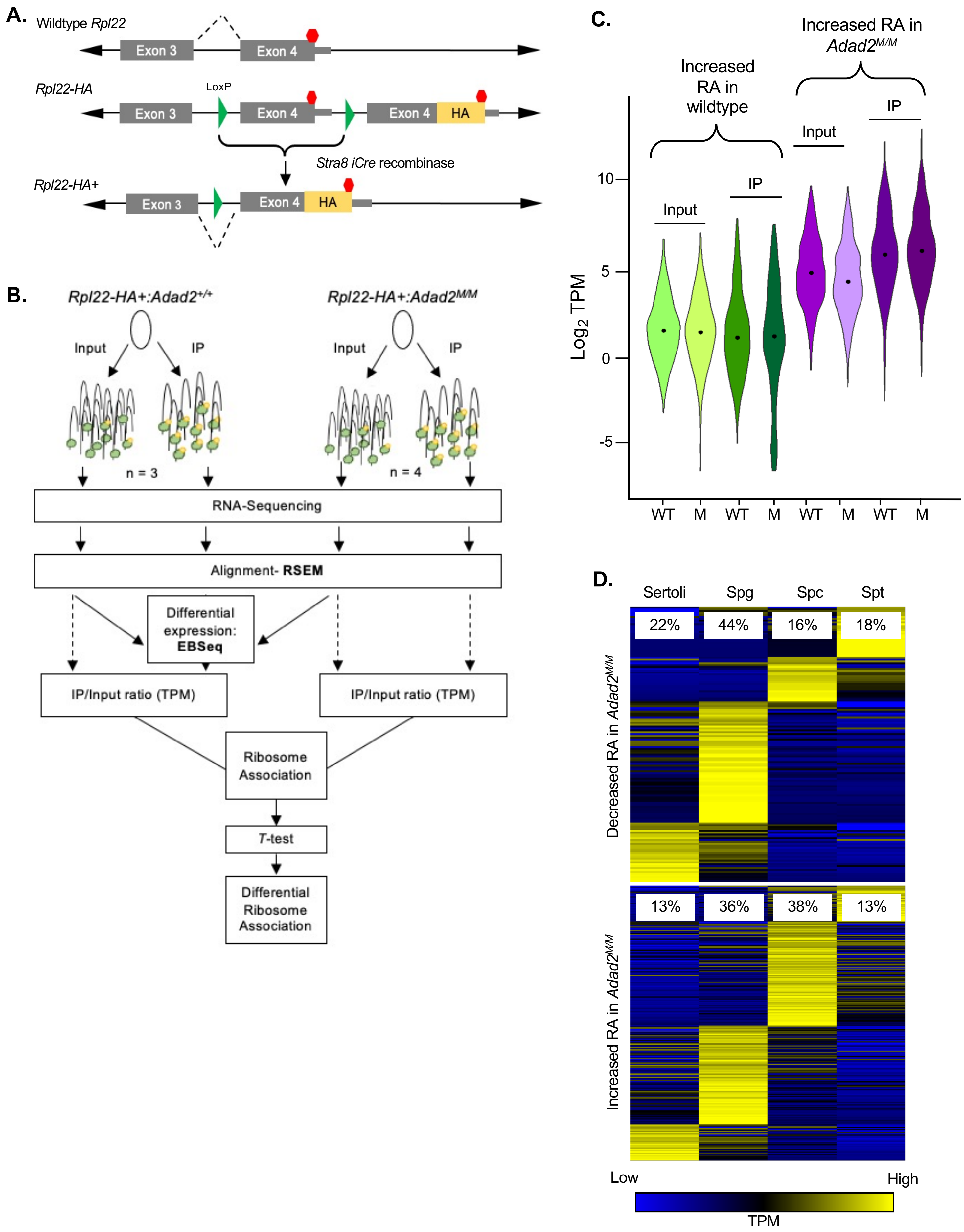

Fig. S4. RiboTag analysis in *Adad2* mutants demonstrates a cell type-specific impact. A) RiboTag scheme showing how Cre expression drives HA-tagged RPL22 by replacing the endogenous *Rpl22* exon 4 with an HA-tagged exon Adapted from Chukrallah et al., 2020. B) Sample generation and data processing for RiboTag. C) Overall abundance of wildtype- or mutant-enriched DRA transcripts across all RNA-sequencing samples shows transcripts with increased RA in mutants tend to be expressed at higher levels in both input and IP (WT – wildtype, M –Adad2M/M). D) Expression of differential ribosome associated genes in isolated wildtype cells (data derived from cell-specific RNA-sequencing datasets (Soumillon et al., 2013)) demonstrates the largest portion of genes with increased RA in the mutant are expressed in meiotic cells. Sertoli - Sertoli cell, Spg –spermatogonia, Spc – Spermatocyte, Spt – spermatid.

Journal of Cell Science • Supplementary information

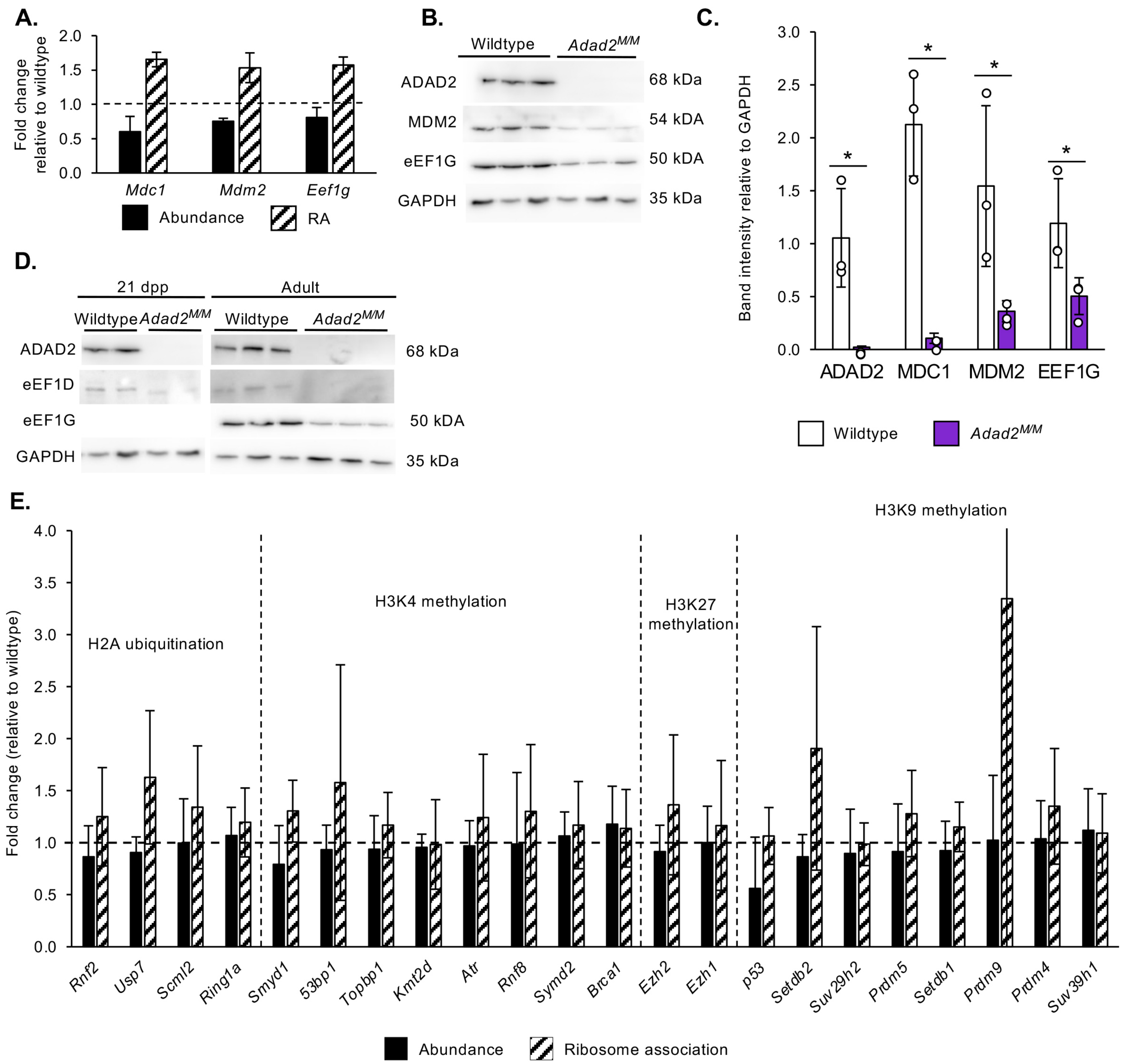

Fig. S5. Altered ribosome association similar to Mdc1 is observed in multiple transcripts but abundance and ribosome association is unaltered in heterochromatin remodeling genes in Adad2 mutants. A) Additional transcripts (Mdm2 and Eef1g) have abundance and ribosome association profiles similar to Mdc1. Dotted line indicates wildtype mean. **B)** Western blot against MDM2 and eEF1G in 21 dpp wildtype and mutant testes and **C)** quantification of band intensity in B shows similar reduction of protein in spite of increased ribosome association. ADAD2 shown as genetic control (n = 3 / genotype). The ADAD2 genotype control and GAPDH loading control blots are also shown for the same protein sample panel in Fig. 1D, Fig. 3A, Fig. S5D and Fig. S6D. Plotted points represent individual samples. **D)** Western blot against eEF1G and eEF1D in adult, and against eEF1D in 21 dpp wildtype and mutant testes (adult, n=3/genotype; 21 dpp, n=2/genotype). Abundance of eEF1G in 21 dpp wildtype and mutant testes can be seen in B. The ADAD2 genotype control blots and GAPDH control blots are also shown for the same protein sample panels in Fig. 1D, Fig. 3A, Fig. S5B and Fig. S6D. **E)** Abundance and ribosome association of transcripts encoding epigenetic regulators of H2A ubiquitination, H3K4 methylation, H3K27 methylation, and H3K9 methylation in Adad2 mutants demonstrating minimal change in either measure. Dotted line indicates wildtype mean.

Journal of Cell Science • Supplementary information

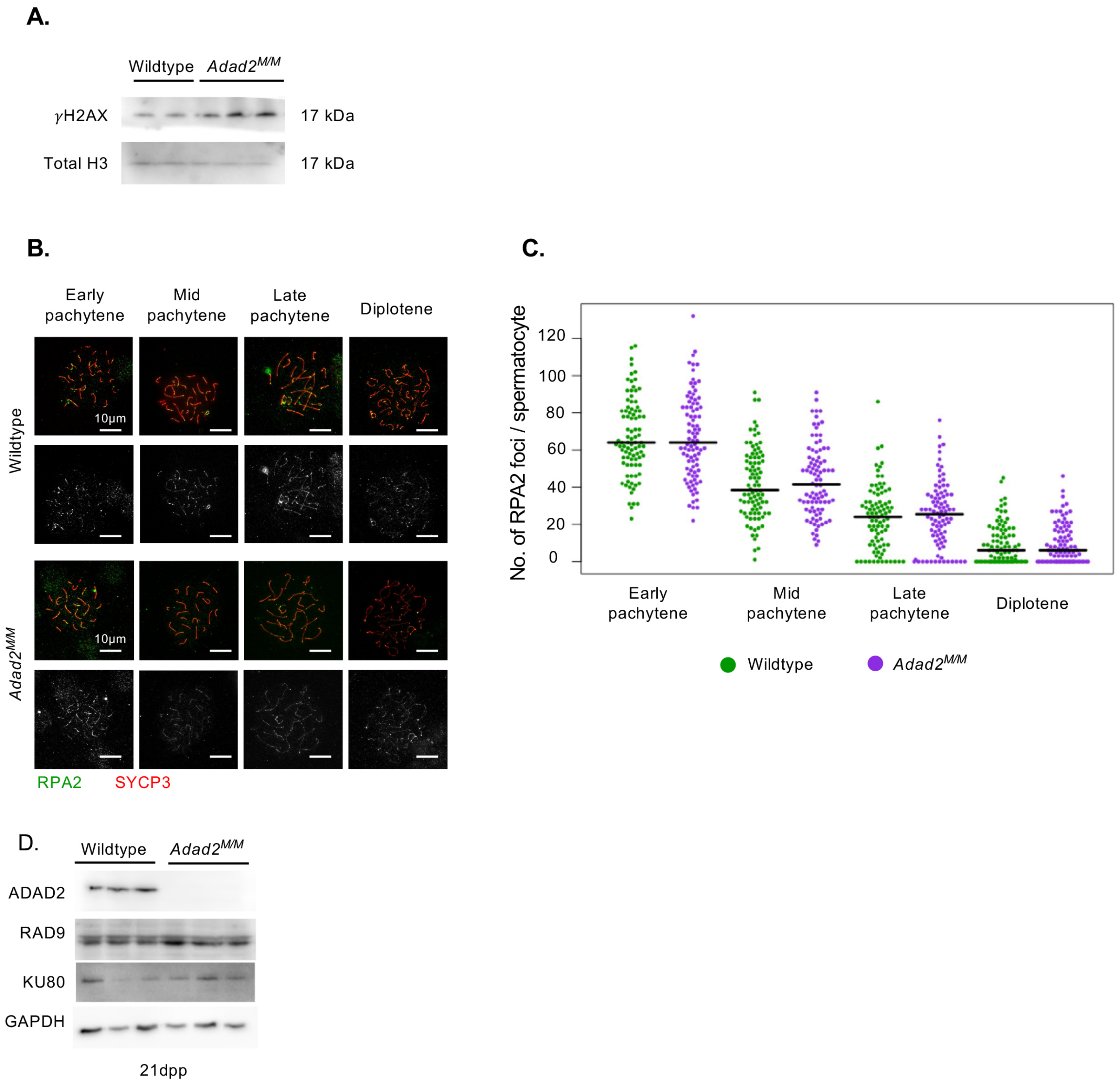

Fig. S6. *Adad2$^{M/M}$* spermatocytes show no evidence of aberrant DNA damage in spite of elevated γH2AX.

A) Western blot of 21 dpp whole testis histone lysate from wildtype and *Adad2$^{M/M}$* samples (wildtype n = 2; *Adad2$^{M/M}$* n = 3) showing increased γH2AX. Total H3 shown as loading control. The total H3 loading control blot is also shown for the same protein sample panel in Fig. 1E. **B)** RPA2 foci images (40x magnification; green - RPA2, red -SYCP3) and **C)** quantification (n = 90 total cells per genotype per cell stage, pooled from n = 3 wildtype and *Adad2$^{M/M}$* samples) shows normal DNA repair kinetics in mutant spermatocytes. Bar represents mean value. **D)** Western blot against RAD9 and KU80 in 21 dpp wildtype and mutant testes showing lack of DNA damage repair pathway induction. ADAD2 and GAPDH shown as genotype and loading controls, respectively. The ADAD2 genotype control and GAPDH loading control blots are also shown for the same protein sample panel in Fig. 1D, Fig. 3A and Fig. S5B,D.

Journal of Cell Science • Supplementary information

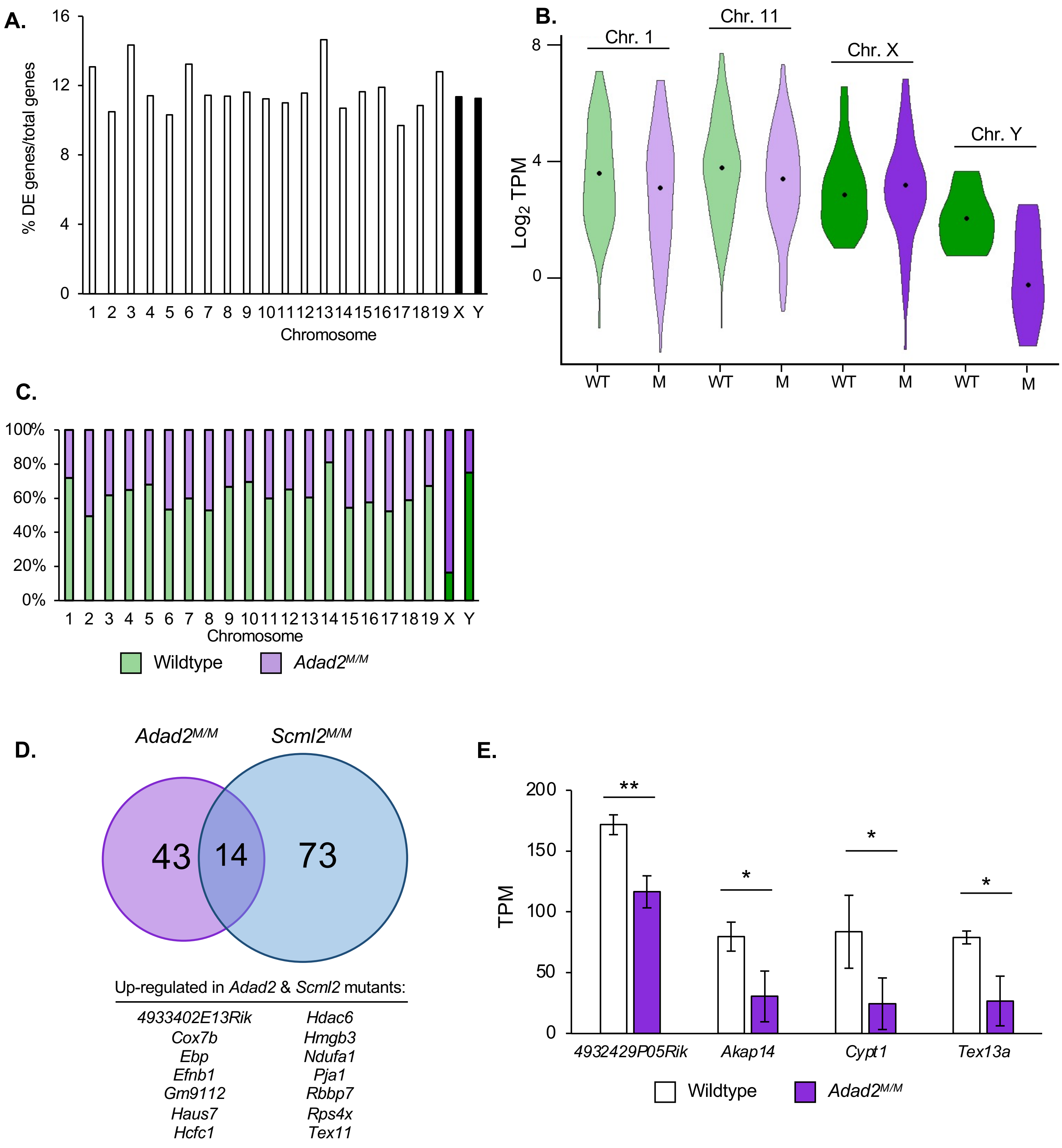

Fig. S7. *Adad2* mutants display moderate X-chromosome gene expression abnormalities. A) Percent of DE genes/total detected on each chromosome in *Adad2*$^{M/M}$ samples showing X and Y chromosomes (in black) are not over-represented in differentially expressed genes. **B)** Gene expression across select autosomes and the sex chromosomes showing the X chromosome does not display a general increase of gene expression in *Adad2* mutants. **C)** Ratio of DE genes on each chromosome that are upregulated in the wildtype (green) or upregulated in the mutant (purple) demonstrating a preponderance of up-regulated X chromosome genes in the mutant. **D)** Venn diagram and list of X chromosome genes up-regulated in *Adad2* and *Scml2* mutants. **E)** Expression of X-encoded de novo MSCI-escaping genes differentially expressed in *Adad2* mutants showing reduced expression in mutants. Error bars represent one standard deviation. Significance calculated using Student's *t*-test. * $p < 0.05$, ** $p < 0.001$, *** $p < 0.0001$

Journal of Cell Science • Supplementary information

A.

Antibody	Species	Source	Reference number	IF conditions	ICC	Western blot conditions
53BP1	Rabbit	Novus	NB100-304	PFA, Tris EDTA Antigen retrieval, 1:500	NA	8% gel, 1:500
Alexafluor Goat anti Mouse 488	Goat	Invitrogen	A11001	1:1000	1:1000	NA
Alexafluor Goat anti Mouse 594	Goat	Invitrogen	A21125	1:1000	1:1000	NA
Alexafluor Goat anti Rabbit 488	Goat	Invitrogen	A11008	1:1000	1:1000	NA
Alexafluor Goat anti Rabbit 594	Goat	Invitrogen	A11012	1:1000	1:1000	NA
Alexafluor Goat anti Rat 488	Goat	Abcam	ab150157	NA	1:1000	NA
Anti-ADAD2 93Term	Rabbit	Snyder et al., 2020	NA	PFA, Citrate antigen retrieval, 1:100	NA	10% gel, 1:1000
Anti-ADAD2 94AP	Rabbit	Snyder et al., 2020	NA	NA	NA	10% gel, 1:1000
ATR	Rabbit	Cell Signalling Technology	2790S	NA	1:300, 37C primary	NA
BRCA1	Rabbit	Abcam	ab90528	PFA, Tris EDTA Antigen retrieval, 1:500	NA	8% gel, 1:500
Donkey anti goat 594	Donkey	Abcam	ab150136	1:1000	1:1000	NA
Donkey anti goat HRP	Goat	Abcam	ab97110	NA	NA	1:2000
Donkey anti Rabbit	Donkey	Abcam	ab150073	1:1000	1:1000	NA
Donkey anti sheep 488	Donkey	Abcam	ab150177	1:1000	1:1000	NA
Donkey anti sheep HRP	Donkey	Abcam	ab6900	NA	NA	1:2000
EEF1D	Rabbit	Abcam	ab153728	NA	NA	10% gel, 1:5000
EEF1G	Rabbit	Abcam	ab72368	NA	NA	10% gel, 1:500
GAPDH	Rabbit	Cell Signalling Technology	14C10	NA	NA	10% gel, 1:500
Goat anti Mouse HRP	Goat	BioRad	172-1011	NA	NA	1:2000
Goat anti Rabbit HRP	Goat	BioRad	172-1019	NA	NA	1:2000
H2AK119ub	Rabbit	Cell Signalling Technology	8240S	NA	1:200	NA
γH2AX	Mouse	MilliporeSigma	05-636	PFA, Tris EDTA Antigen retrieval, 1:100	1:100	Histone extraction, 15% gel, 1:500
H3K27me3	Rabbit	Gift from William Belden	NA	NA	NA	Histone extraction, 15% gel, 1:500
H3K27me3	Mouse	ActiveMotif	AB_2793246	PFA, Tris EDTA Antigen retrieval, 1:100	NA	NA
H3K4me2	Rabbit	MilliporeSigma	07-030	PFA, Tris EDTA Antigen retrieval, 1:200	NA	Histone extraction, 15% gel, 1:500
H3K9me3	Rabbit	MilliporeSigma	07-442	PFA, Tris EDTA Antigen retrieval, 1:200	NA	Histone extraction, 15% gel, 1:500
HP1α	Goat	Abcam	ab77256	NA	NA	15% gel, 1:1000
HP1β	Rabbit	Proteintech	10241-2-AP	NA	NA	15% gel, 1:1000
HP1γ	Rabbit	Abcam	ab66617	NA	NA	15% gel, 1:1000
Ku80	Rabbit	Abcam	ab80592	NA	NA	10% gel, 1:1000
MDC1	Sheep	BioRad	AHP799	PFA, Tris EDTA Antigen retrieval, 1:100	NA	NA
MDC1	Rabbit	Novus	NB100-395	NA	NA	10% gel, 1:1000
MDM2	Rabbit	Abcam	ab16895	NA	NA	10% gel, 1:1000
Pan H3	Rabbit	MilliporeSigma	06-755	NA	NA	Histone extraction, 15% gel, 1:500
RAD51	Rabbit	Novus	NB100-148	NA	NA	10% gel, 1:1000
RAD9	Rabbit	Genetex	gtx100078	NA	NA	10% gel, 1:500
RPA2	Rat	Cell Signalling Technology	2208S	NA	1:500, 4C primary	NA
SYCP3	Mouse	Abcam	ab97672	PFA, Tris EDTA Antigen retrieval, 1:300	1:200	NA
SYCP3	Rabbit	Novus	NB300-232	PFA, Tris EDTA Antigen retrieval, 1:300	1:200	NA
TNP1	Rabbit	Proteintech	17178-1-AP	PFA, Tris EDTA Antigen retrieval, 1:500	NA	NA
USP7	Rabbit	Bethyl	A300-033A	NA	1:300	NA

B.

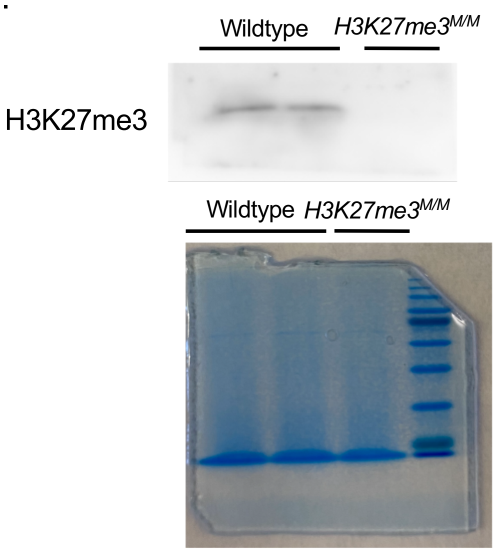

Fig. S8. Sourcing and validation of antibodies. A. Antibodies utilized throughout this manuscript. Sources and concentrations by application included for all. **B.** Western blot of H3K27me3 antibody used for western blotting in histone lysate from wildtype (n = 2) and *H3K27me3*$^{M/M}$ (n = 1) extracted from neurospora. Histone lysate from wildtype (n = 2) and *H3K27me3*$^{M/M}$ (n = 1) loaded gel stained with Coomassie to validate loading.

Journal of Cell Science • Supplementary information

Table S1. Genes with differential ribosome association in *Adad2* mutants.
List of differentially ribosome associated (DRA) genes as determined by calculating the ratio of IP TPM over input TPM. Any transcripts with any input values equaling zero were removed. A Welch t-test was conducted using R to identify differential ribosome association (DRA, $p < 0.05$)

Click here to download Table S1

Table S2. Differentially expressed genes in *Adad2* mutants.
List of differentially expressed genes as identified via EBSeq. PPEE: posterior probability of equal expression, PPDE posterior probability of differential expression, PostFC- posterior fold change and RealFC: true fold change.

Click here to download Table S2
