# Supplementary material for: ADAD2 regulates heterochromatin in meiotic and post-meiotic male germ cells via translation of MDC1
Source: J Cell Sci. 2022 Feb 22;135(4):jcs259196. doi: 10.1242/jcs.259196 (PMC8919335; doi:10.1242/jcs.259196)
Supplement: Supplementary file 1 [file joces-135-259196_review_history.pdf]

ADAD2 regulates heterochromatin in meiotic and post-meiotic male germ cells via translation of MDC1

Lauren G. Chukrallah, Aditi Badrinath, Gabrielle G. Vittor and Elizabeth M. Snyder
DOI: 10.1242/jcs.259196

Editor: Maria Carmo-Fonseca

Review timeline
Original submission: 29 July 2021
Editorial decision: 27 September 2021
First revision received: 10 December 2021
Accepted: 9 January 2022

Original submission

First decision letter

MS ID#: JOCES/2021/259196

MS TITLE: ADAD2 regulates heterochromatin in meiotic and post-meiotic male germ cells via translation of MDC1

AUTHORS: Lauren G Chukrallah, Aditi Badrinath, Gabrielle G Vittor, and Elizabeth M Snyder
ARTICLE TYPE: Research Article

We have now reached a decision on the above manuscript.

To see the reviewers' reports and a copy of this decision letter, please go to: https://submit-jcs.biologists.org and click on the 'Manuscripts with Decisions' queue in the Author Area. (Corresponding author only has access to reviews.)

As you will see, the reviewers raise a number of substantial criticisms that prevent me from accepting the paper at this stage. They suggest, however, that a revised version might prove acceptable, if you can address their concerns. If you think that you can deal satisfactorily with the criticisms on revision, I would be pleased to see a revised manuscript. We would then return it to the reviewers.

We are aware that you may be experiencing disruption to the normal running of your lab that makes experimental revisions challenging. If it would be helpful, we encourage you to contact us to discuss your revision in greater detail. Please send us a point-by-point response indicating where you are able to address concerns raised (either experimentally or by changes to the text) and where you will not be able to do so within the normal timeframe of a revision. We will then provide further guidance. Please also note that we are happy to extend revision timeframes as necessary.

Please ensure that you clearly highlight all changes made in the revised manuscript. Please avoid using 'Tracked changes' in Word files as these are lost in PDF conversion.

I should be grateful if you would also provide a point-by-point response detailing how you have dealt with the points raised by the reviewers in the 'Response to Reviewers' box. Please attend to all of the reviewers' comments. If you do not agree with any of their criticisms or suggestions please explain clearly why this is so.

© 2022. Published by The Company of Biologists under the terms of the Creative Commons Attribution License (https://creativecommons.org/licenses/by/4.0/).

Reviewer 1

Advance summary and potential significance to field

This manuscript reports the function of ADAD2, a testis-specific adenosine deaminase (AD) domain containing protein, on heterochromatin formation in spermatocytes and spermatids through MDC1 translation.
Previously, the authors have reported that germ cells of Adad2 mutants do not progress beyond round spermatid (Snyder et al., 2020). In the present manuscript, they analyzed heterochromatin levels (Fig.1) and genes of ribosome association (Fig. 2), and then identified BRCA1 and/or MDC1 as potential mediator of ADAD2 (Fig. 3). Although increased BRCA1 was observed in the mutant, after analyses of XY-body ATR and gammaH2AX the authors concluded increased BRCA1 had minimal impact on their behavior (Fig. 4). On the other hand, they observed that MDC-dependent enrichment of the deubiquitinating enzyme USP7 and exclusion of the epigenetic mark K119Ub were reduced in XY-body of the mutant (Fig. 5). Furthermore H3K4me2 euchromatin marker, which is dependent on the MDC1-interacting RNF8, was not observed in XY-body of the mutant (Fig. 6). They also showed that Adad2 mutant spermatids have abnormalities in post-meiotic chromatin and chromatin remodeling by analyses of the heterochromatin markers, H3K27me3 and H3K9me3, and H3K4me2 (Fig. 7). From these results, the authors conclude that ADAD2 has a function on Mdc2 translation (Fig. 8).
The present manuscript seems to provide a new insight of ADAD2 functions, but unfortunately it is difficult for me to decide whether authors' claim is appropriate by following reasons. First, the authors have not provided any data how ADAD2 regulates Mdc1 translation. Fig. 2 showed that abundance Mdc1 mRNA decreased but the ribosome association increased. Fig. 3 showed decline of MDC1 protein in the mutant.
Further experiments for these phenomena will be needed. Second, most heterochromatin status by Western blot were from testis lysate, except H3K4me2 in Fig. 6. Quantitative analyses of other markers in spermatocytes are necessary. Third, no rationale for % on the vertical axis in Fig. 4-6 shown. Please describe what and how many samples were analyzed. Fourth, Supplemental Figures 3 and 5 are same, and I could not find Supplemental Figure 7. Therefore, I could not evaluate their description (L290, L301, L306, L310, L318 L331).

Comments for the author

This manuscript reports the function of ADAD2, a testis-specific adenosine deaminase (AD) domain containing protein, on heterochromatin formation in spermatocytes and spermatids through MDC1 translation. Previously, the authors have reported that germ cells of Adad2 mutants do not progress beyond round spermatid (Snyder et al., 2020). In the present manuscript, they analyzed heterochromatin levels (Fig.1) and genes of ribosome association (Fig. 2), and then identified BRCA1 and/or MDC1 as potential mediator of ADAD2 (Fig. 3). Although increased BRCA1 was observed in the mutant, after analyses of XY-body ATR and γH2AX the authors concluded increased BRCA1 had minimal impact on their behavior (Fig. 4). On the other hand, they observed that MDC-dependent enrichment of the deubiquitinating enzyme USP7 and exclusion of the epigenetic mark K119Ub were reduced in XY-body of the mutant (Fig. 5). Furthermore, H3K4me2 euchromatin marker, which is dependent on the MDC1-interacting RNF8, was not observed in XY-body of the mutant (Fig. 6). They also showed that Adad2 mutant spermatids have abnormalities in post-meiotic chromatin and chromatin remodeling by analyses of the heterochromatin markers, H3K27me3 and H3K9me3, and H3K4me2 (Fig. 7). From these results, the authors conclude that ADAD2 has a function on *Mdc2* translation (Fig. 8).
The present manuscript seems to provide a new insight of ADAD2 functions, but unfortunately it is difficult for me to decide whether authors' claim is appropriate by following reasons. First, the authors have not provided any data how ADAD2 regulates *Mdc1* translation. Fig. 2 showed that abundance *Mdc1* mRNA decreased but the ribosome association increased. Fig. 3 showed decline of MDC1 protein in the mutant. Further experiments for these phenomena will be needed. Second, most heterochromatin status by Western blot were from testis lysate, except H3K4me2 in Fig. 6. Quantitative analyses of other markers in spermatocytes are necessary. Third, no rationale for % on the vertical axis in Fig. 4-6 shown. Please describe what and how many samples were analyzed. Fourth, Supplemental Figures 3 and 5 are same, and I could not find Supplemental Figure 7. Therefore, I could not evaluate their description (L290, L301, L306, L310, L318, L331).

© 2022. Published by The Company of Biologists under the terms of the Creative Commons Attribution License (https://creativecommons.org/licenses/by/4.0/).

In addition, this manuscript is not easy to read probably because the story goes over and over, and the result is not written clearly enough. I hope authors revise it.

Minor comments
Please describe developmental stages of germ cells in 21, 25 and 30 dpp.
L225. "normally express high level of granule-localized ADAD2" needs the reference.

L279. Is Fig. 5B 5A?

Fig. 3. Please explain stages IV, VII, IX, and XII. Why are expression patterns of SYCP3 different between B and C in wildtype? Inset lines disturb to see signals.

Fig. 7. Please indicate where to look. Why do H3K27me3 signals in the mutant look different between A and B? Inset lines disturb to see signals.

Reviewer 2

Advance summary and potential significance to field

This manuscript does an excellent job demonstrating the significant role of ADAD2 for MDC1 translation and the role of MDC1 in maintaining the meiotic male germ cell heterochromatin. The article highlights important data that ADAD2 mutants leads to defective MDC1 translation, which rise to aberrant heterochromatin in both autosomes and the sex chromosomes of late meiotic spermatocytes.
I was delighted to see how they explained the mechanisms of germ cell death in ADAD2 mutants and highpoint the central role of MDC1 in maintaining heterochromatin in both chromatin compartments of the late meiotic germ cell and post-meiotic germ cell.
I also like the way they presented their figures, but I was a little disappointed that the western blots in figure one are not clear to illustrate the significant increase of HP1α (c) or heterochromatin increasing by H3K9me3 and H3K27me3, and euchromatin decreasing by H3K4me2 (d), so it should be repeated with high specific antibodies and avoid background.
Overall, this paper highly meets the journal criteria and it should be accepted for publication.

Comments for the author

This manuscript does an excellent job demonstrating the significant role of ADAD2 for MDC1 translation and the role of MDC1 in maintaining the meiotic male germ cell heterochromatin. The article highlights important data that ADAD2 mutants leads to defective MDC1 translation, which rise to aberrant heterochromatin in both autosomes and the sex chromosomes of late meiotic spermatocytes.
I was delighted to see how they explained the mechanisms of germ cell death in ADAD2 mutants and highpoint the central role of MDC1 in maintaining heterochromatin in both chromatin compartments of the late meiotic germ cell and post-meiotic germ cell.
I also like the way they presented their figures, but I was a little disappointed that the western blots in figure one are not clear to illustrate the significant increase of HP1α (c) or heterochromatin increasing by H3K9me3 and H3K27me3, and euchromatin decreasing by H3K4me2 (d), so it should be repeated with high specific antibodies and avoid background.
Overall, this paper highly meets the journal criteria and it should be accepted for publication.

Reviewer 3

Advance summary and potential significance to field

In this manuscript Lauren G. Chukrallah et a., describes how ADAD2 regulates meiotic and post-meiotic heterochromatin via MDC1. The authors demonstrate that maintenance of MSCI that is established early in meiosis, is key to proper differentiation of post-meiotc germ cells.

© 2022. Published by The Company of Biologists under the terms of the Creative Commons Attribution License (https://creativecommons.org/licenses/by/4.0/).

This is a very interesting work that answers a relevant biological question in the field. The manuscript is well written, and most conclusions straight forward and supported by the experimental results. There is no doubt it deserves publication.
However, I have some concerns listed below that need to be addressed point, by point.

Comments for the author

I encourage revision. Please respond point, by point.

Major concerns:
Fig. 1A: Round spermatides (Rs) with 3+ foci are present in a relatively restrict population of round spermatids (less than 20%). Is it because most Rs have been already eliminated by apoptosis? If so, please repeat the experiment at a time point just before a massive elimination of Rs occurs. 20% doesn't seems to be reasonable biologically relevant.
Fig. 1C. By comparing the WB analysis with quantification, it seems not to be a match between the image and the graph. HP1α expression is much stronger in Adad2M/M mice, than HP1ß and HP1γ. This is not the case in the graph at the side.
Please, use images that are representative of quantification.
Row 132. To conclude that mutant round spermatids truly have an altered chromatin state, you must proof it using enriched populations. Purified fractions of Rs can be obtained by either centrifugal elutriation, FACS sorting or STA-PUT velocity sedimentation. The latter allows isolation of purified fractions also from testis of a single mouse.
Row 214. There is NO SF3A-C in the manuscript. SF3A-C and SF5 are identical.
Row 2019. This mean that you cannot exclude that the Adad2-null phenotype is ascribed to deregulation of other protein factors. There is not much mention on that, especially in the discussion. Authors logically focus on MDC1 but they should discuss data in a broader context.
Fig. 3B. Staging is not convincing. Please, explain in detail how stages IV, VII and IX are univocally identified, in absence of normal germ cells association (mutant) and of stage-specific markers. Since γH2ax pattern is qualitatively not altered in the mutant, one way could be to use it to stage tubules in association with SYCP3 (see for instance Supplementary material in Di Giacomo et al., Mol Cell 2013. DOI 10.1016/j.molcel.2013.04.026).
Row 226. Figure 3C (stage VII) is not representative of what is stated in the text. Intensity of MDC1 in the sex body at stage VII is as low as that in stage XII. Moreover, stage XII is supposed to contain zygotene-stage spermatocytes, not late pachytene stage cells with sex body. Thus, you are not looking at the right cell type. A detailed analysis of MDC1 in mutat is lacking. Please, show what the pattern of MDC1 is, using chromosome spreads. Show MDC1 localization, level and spreading over the sex chromatin.
Fig. 4B. Images are NOT representative of what shown in the graph. Levels of ATR in mid-pachynema in the mutant is much higher than what shown in the graph.
Moreover, in the graph on abscissa you wrote "autosomal ATR". In principle it is correct if you refer to cells where XY chromosomes are identified, and you have excluded ATR-associated to XY from the measure. In the mutant, this is likely doable in cells from late-pachytene onward, when XY are visible, not in early to mid-pachytene stage, where XY-associated ATR cannot be distinguished from that associated with the autosomes. Please, explain in detail how measurement of ATR signal onto autosomes was performed, excluding ATR signal associated to XY chromosomes.
Row 255. There is not persistent association of ATR in late-pachytene stage cells while phosphorylatin of H2AX persists. How do you reconcile this result with your interpretation? Authors should check persistence of DSBs using one additional DSB repair marker such as DMC1. According to Testa et al., JCS 2018 (DOI 10.1242/jcs.214411), a lack of MDC1 reduces the stringency of the recombination-dependent checkpoint and it allows progression to mid-pachynema of cells with unrepaired DSBs (i.e. with greater number of DMC1 foci). Thus, the increased γH2AX level at late-pachynema might be linked to the persistence of DSBs intermediates that are not marked by RPA.

Minor concerns:
Please provide a table with all primary and secondary antibodies used in the study, including source, catalog number, dilutions and application.
Row 18. The first demonstration that failure in XY body formation results in meiotic cells arrest comes from the studies on the role of histone H2AFX in meiosis. The reference below should thus be included:

© 2022. Published by The Company of Biologists under the terms of the Creative Commons Attribution License (https://creativecommons.org/licenses/by/4.0/).

• Fernandez-Capetillo et al., H2AX is required for chromatin remodeling and inactivation of sex chromosomes in male mouse meiosis, Dev Cell, 2003. PMID 12689589 Row 43. In addition to MSCI, MDC1 is also involved in controlling recombination
(MLH3 assembly) and the activation of the recombination-dependent checkpoint. The following reference should be included in the list:
• Testa et al., H2AFX and MDC1 promote maintenance of genomic integrity in male germ cells, JCS 2018. DOI 10.1242/jcs.214411 Fig.1B. It would be good to have Rs identified by H1t staining. Alternatively please indicate whether you are looking at the same stage of the epithelial cell cycle in wt Vs Adad2M/M, and identify the stage.
Row 115. 1B should be 1C Fig. 1C. Statistical significance of HP1α expression in adult is limited. This is likely due to the fact you are using total testis extracts. To increase significance of Hp1α expression, I suggest performing western blotting from isolated fractions of Rs.
Fig. 1D. Again, to increase significance in methylation pattern changes, I suggest performing western blotting from isolated fractions of round spermatids.
Row 145. It is not clear to me how data shown in S1A have been obtained. Have you isolated and analyzed wild type cells, or you just assigned DE genes to each testis cell category accordingly to data in literature?
Fig. S1E-F. How have you identified stages of the epithelial cell cycle? It is very hard to do it by just using DAPI stained sections. Lack of post meiotic germ cells in the mutant makes identification of stages even more difficult. Please explain procedure in detail.
Fig. 7C. Please provide evidence of antibody staining specificity in elongated spermatids by blocking with a TNP1 peptide and showing in supplementary the staining pattern with just secondary antibodies.

First revision

Author response to reviewers' comments

To whom it may concern,

The authors would like to thank the reviewers for their suggestions, which we feel have significantly improved this manuscript and well prepared it for publication. We would especially like to direct reviewers to newly added data exploring the mechanism by which ADAD2 influences translation (Supplemental Figure 4) and confirming protein and histone mark alterations in isolated cell populations (Figure 1). This new data ***further confirms the impacts of ADAD2 action specifically on meiotic spermatocytes and additionally demonstrates ADAD2 likely regulates translation elongation via the eEF1B complex****. We have also resolved upload errors from our original supplemental figures and tables and have included all referenced figures and tables in this revision. Responses to individual comments may be found below in italics. As a result of word restrictions and to enhance clarity, portions of the text have been edited for enhanced clarity. Where this editing impacted content, it has been indicated in blue. For ease of review, all requested revisions can be found below and have been indicated in the revised manuscript. Additionally, to reduce file size, optimized figures have been provided for review. High resolution images are available for publication.*

Sincerely,

The Authors

Reviewer Comments:

Reviewer 1

This manuscript reports the function of ADAD2, a testis-specific adenosine deaminase (AD) domain containing protein, on heterochromatin formation in spermatocytes and spermatids through MDC1 translation. Previously, the authors have reported that germ cells of *Adad2* mutants do not progress beyond round spermatid (Snyder et al., 2020). In the present manuscript, they analyzed

© 2022. Published by The Company of Biologists under the terms of the Creative Commons Attribution License (https://creativecommons.org/licenses/by/4.0/).

heterochromatin levels (Fig.1) and genes of ribosome association (Fig. 2), and then identified BRCA1 and/or MDC1 as potential mediator of ADAD2 (Fig. 3).
Although increased BRCA1 was observed in the mutant, after analyses of XY-body ATR and gammaH2AX the authors concluded increased BRCA1 had minimal impact on their behavior (Fig. 4). On the other hand, they observed that MDC-dependent enrichment of the deubiquitinating enzyme USP7 and exclusion of the epigenetic mark K119Ub were reduced in XY-body of the mutant (Fig. 5). Furthermore, H3K4me2 euchromatin marker, which is dependent on the MDC1-interacting RNF8, was not observed in XY-body of the mutant (Fig. 6). They also showed that Adad2 mutant spermatids have abnormalities in post-meiotic chromatin and chromatin remodeling by analyses of the heterochromatin markers, H3K27me3 and H3K9me3, and H3K4me2 (Fig. 7). From these results, the authors conclude that ADAD2 has a function on Mdc2 translation (Fig. 8). The present manuscript seems to provide a new insight of ADAD2 functions, but unfortunately it is difficult for me to decide whether authors' claim is appropriate by following reasons.

The present manuscript seems to provide a new insight of ADAD2 functions, but unfortunately it is difficult for me to decide whether authors' claim is appropriate by following reasons.

1. First, the authors have not provided any data how ADAD2 regulates Mdc1 translation. Fig. 2 showed that abundance Mdc1 mRNA decreased but the ribosome association increased. Fig. 3 showed decline of MDC1 protein in the mutant. Further experiments for these phenomena will be needed.

We wholly agree with this reviewer's assessment and as such have added several additional analyses aimed to identify a potential molecular function of ADAD2. In short, we show Adad2 mutants have reduction of the eEF1B complex, which is required for normal translation elongation (see full manuscript text below, in blue). In other systems where translation elongation is reduced, transcripts show 1) enhanced ribosome association, 2) moderate reduction in transcript abundance, and 3) dramatic loss of protein. This pattern mimics what is observed for Mdc1 in Adad2 and indicates ADAD2 is a potential regulator of Mdc1 translation elongation via the eEF1B complex. Current efforts, to be described in future reports, are underway to define the exact influence of ADAD2 on the eEF1B complex.

> **Increased ribosome association is normally assumed to be indicative of increased protein production. However increased ribosome occupancy can lead to reduced protein production and subsequent transcript degradation in cases of either ribosome stalling or reduced translation elongation (Brandman et al., 2012; D'Orazio et al., 2019). Transcript abundance and ribosome association of ribosome stress response transcripts were not substantially altered in *Adad2* mutants, indicating no widespread ribosome stalling. As such, we next focused on regulators of translation elongation. Translation elongation requires both the eEF1A complex which delivers an amino acid charged tRNA to the ribosome and the eEF1B complex which contains a guanine nucleotide exchange factor (GEF) required for eEF1A activity (Sasikumar et al., 2012). Our previous analysis showed eEF1G, a structural component of the eEF1B complex, had reduced protein abundance in *Adad2* mutants (SF 5B). Thus, we quantified the abundance of eEF1D, one of two eEF1A GEFs in the eEF1B complex, to determine whether eEF1B complex functionality may be reduced in *Adad2* mutants (SF 5D). This analysis demonstrated significant reduction of total eEF1D in both 21 dpp and adult testis. These changes mirror those observed for eEF1G and demonstrate reduction of the eEF1B complex in *Adad2* mutants. This, together with our analyses of the *Mdc1* transcript and protein, implicate ADAD2 as a regulator of translation elongation in meiotic germ cells and suggest abnormal translation elongation of key regulatory proteins may underpin the *Adad2* phenotype.**

© 2022. Published by The Company of Biologists under the terms of the Creative Commons Attribution License (https://creativecommons.org/licenses/by/4.0/).

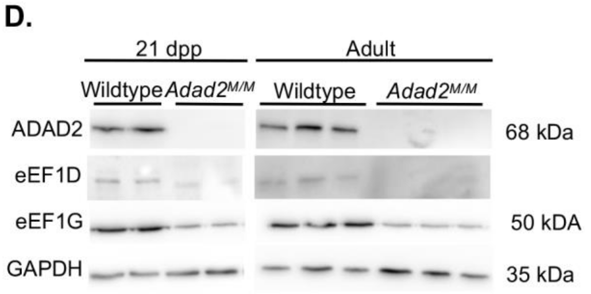

2. Second, most heterochromatin status by Western blot were from testis lysate, except H3K4me2 in Fig. 6. Quantitative analyses of other markers in spermatocytes are necessary.

We thank the reviewer for this suggestion and have several additional analyses that we hope will address the concern. Foremost, in order to confirm heterochromatin state in specific cell populations, we have examined H3K9me3, H3K27me3, H3K4me2, along with all three HP1 proteins in cell-type enriched protein isolations. These analyses are included as additional panels in Figure 1D and E and the cell-type enrichment values reported in newly added Supplemental Figure 1B. We hope these additional analyses, in combination with our previous reported 1) quantification of spermatocyte number (now Supplemental Figure 3B) showing no loss of spermatocytes in mutant testes and 2) TUNEL analysis (now Supplemental Figure 2D) showing no increased apoptosis in mutant spermatocytes provide sufficient support for our supposition that heterochromatin status (as assessed by Western blotting) is abnormal in mutant spermatocytes.

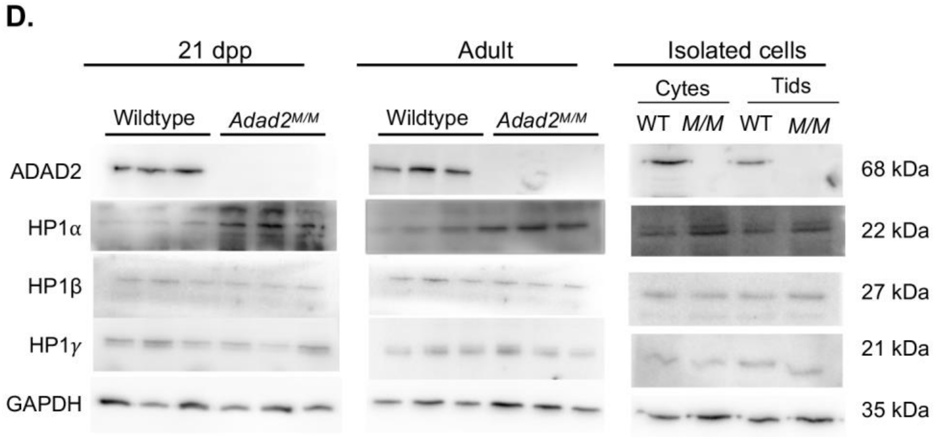

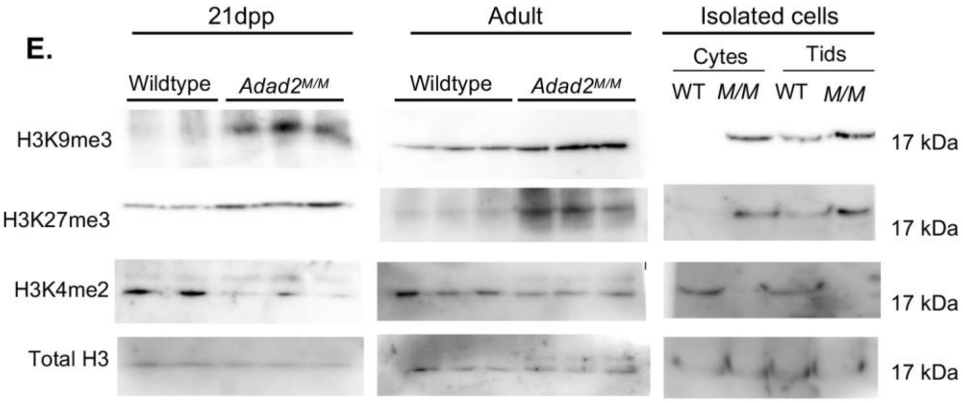

3. Third, no rationale for % on the vertical axis in Fig. 4-6 shown. Please describe what and how many samples were analyzed.

© 2022. Published by The Company of Biologists under the terms of the Creative Commons Attribution License (https://creativecommons.org/licenses/by/4.0/).

Thank you for pointing out this gap in our presentation. In short, axes represent the frequency specific immunocytochemical staining patterns within a given cell type. For example, Fig. 4A reports the percentage of early, mid, and late pachytenes with autosomal gH2AX in both wildtype and mutant. Biological justification and description for these quantifications has been added to the results section, axes within the figures relabeled, and sample descriptions added to both the figure legends and methods.

4. Fourth, Supplemental Figures 3 and 5 are same, and I could not find Supplemental Figure 7. Therefore, I could not evaluate their description (L290, L301, L306, L310, L318, L331).

Thank you for letting us know about this error. We have taken special pains to ensure all supplemental figures and tables have been provided correctly.

5. In addition, this manuscript is not easy to read probably because the story goes over and over, and the result is not written clearly enough. I hope authors revise it.

Thank you for your advice. The manuscript has been edited for readability and clarity as needed.

Minor comments

1. Please describe developmental stages of germ cells in 21, 25 and 30 dpp.

A detailed description of the cellular composition of the testis at all time points used in this analysis (21 dpp, 30 dpp, 50-70 dpp, and adult) has been added in either the results or the methods. These can be found within the manuscript as well as below, in blue.

21 dpp and adult testis:
The HP1 proteins were examined at two points in testis development: the adult, which contains the full complement of germ cells and whose proteome is dominated by post-meiotic germ cells, and 21 dpp, in which late meiotic germ cells are the most abundant cell population, to estimate when heterochromatin changes may arise in the mutant.

30 dpp:
Testes from 30 dpp animals were used as they contain the full complement of meiotic cells along with the round spermatids of all developmental stages but not elongating or elongated spermatids or spermatozoa.

50-70 dpp
In order to capture each population of interest, testis from 50-70 dpp animals were used. These testes include all stages of meiotic and post-meiotic germ cell development.

2. L225. "normally express high level of granule-localized ADAD2" needs the reference.

The appropriate reference has been added.

3. L279. Is Fig. 5B 5A?

We apologize for the confusion and thank the reviewer for pointing out this issue. The initial reference to Fig. 5 is to direct the reader to the schematic of the signaling cascade upstream of H2K119Ub (Fig. 5A). The second reference to Fig. 5 is to direct the reader to the analysis of USP7 (Fig. 5B) and the last to the analysis of H2K119Ub (Fig. 5C). This has been clarified in the text.

4. Fig. 3. Please explain stages IV, VII, IX, and XII. Why are expression patterns of SYCP3 different between B and C in wildtype? Inset lines disturb to see signals.

The stages described are those defined by Russel et al. (reference added) and refer to the unique cellular associations observed during germ cell differentiation. We have clarified the parameters by which we define stage using DAPI and SYCP3 staining pattern in the newly added Supplemental Figure 1A. Differences between tubules are expected as images are derived from different samples, however in all cases, representative images conform to the expectations set out in our

© 2022. Published by The Company of Biologists under the terms of the Creative Commons Attribution License (https://creativecommons.org/licenses/by/4.0/).

staging paradigm. Additionally, new images and clarification text for Fig. 3B have been added to more accurately represent our observations. Lastly, inset lines have been removed for clarity.

5. Fig. 7. Please indicate where to look. Why do H3K27me3 signals in the mutant look different between A and B? Inset lines disturb to see signals.

Arrows indicating chromocenters and arrowheads indicating PMSC in wildtype round spermatids along with open arrowheads indicating DAPI-rich foci in mutant spermatids have been added to Fig 7A and B. Differences in the H3K27me3 signal is expected given the variable nature of immunofluorescence and the slightly different staining parameters needed for the two different co-immunofluorescent analyses. As noted in both however, H3K27me3 is observed in DAPI-rich foci. Inset lines have been removed as requested.

Reviewer 2 Advance Summary and Potential Significance to Field...

This manuscript does an excellent job demonstrating the significant role of ADAD2 for MDC1 translation and the role of MDC1 in maintaining the meiotic male germ cell heterochromatin. The article highlights important data that ADAD2 mutants leads to defective MDC1 translation, which rise to aberrant heterochromatin in both autosomes and the sex chromosomes of late meiotic spermatocytes. I was delighted to see how they explained the mechanisms of germ cell death in ADAD2 mutants and highpoint the central role of MDC1 in maintaining heterochromatin in both chromatin compartments of the late meiotic germ cell and post-meiotic germ cell. I also like the way they presented their figures, but I was a little disappointed that the western blots in figure one are not clear to illustrate the significant increase of HP1α (c) or heterochromatin increasing by H3K9me3 and H3K27me3, and euchromatin decreasing by H3K4me2 (d), so it should be repeated with high specific antibodies and avoid background. Overall, this paper highly meets the journal criteria and it should be accepted for publication.

Reviewer 2 Comments for the Author...

I was a little disappointed that the western blots in figure one are not clear to illustrate the significant increase of HP1α (c) or heterochromatin increasing by H3K9me3 and H3K27me3, and euchromatin decreasing by H3K4me2 (d), so it should be repeated with high specific antibodies and avoid background.

The authors would very much like to thank Reviewer 2 for their appreciation of this work! To address your concern:

We agree the Western blots are far from perfect. In order to alleviate concerns, we direct Reviewer 2 to Supplemental Figure 8A, which describes antibodies and assay conditions used in this manuscript. As indicated, with the exception of H3K27me3 for western blotting, all antibodies were from commercial sources and commercially validated. For each, extensive efforts were made to select the most appropriate antibody and condition for each assay. In the case of H3K27me3 for western blotting, in house validation using select histone modification mutants demonstrated a high degree of specificity. This validation has been added as new data in Supplemental Figure 8B. Secondly, we have also confirmed our whole testis lysate findings on isolated germ cell populations (newly added panels to Figure 1D and E), further lending support to our original observations of abnormal heterochromatin state in mutant spermatocytes.

Reviewer 3 Advance Summary and Potential Significance to Field...

In this manuscript Lauren G. Chukrallah et a., describes how ADAD2 regulates meiotic and post-meiotic heterochromatin via MDC1. The authors demonstrate that maintenance of MSCI that is established early in meiosis, is key to proper differentiation of post-meiotc germ cells. This is a very interesting work that answers a relevant biological question in the field. The manuscript is well written, and most conclusions straight forward and supported by the experimental results. There is no doubt it deserves publication. However, I have some concerns listed below that need to be addressed point, by point.

I encourage revision. Please respond point, by point.

© 2022. Published by The Company of Biologists under the terms of the Creative Commons Attribution License (https://creativecommons.org/licenses/by/4.0/).

We would like to thank Reviewer 3 for their thoughtful review and helpful recommendations. We have addressed them point by point below.

Major concerns:

1. Fig. 1A: Round spermatides (Rs) with 3+ foci are present in a relatively restrict population of round spermatids (less than 20%). Is it because most Rs have been already eliminated by apoptosis? If so, please repeat the experiment at a time point just before a massive elimination of Rs occurs. 20% doesn't seems to be reasonable biologically relevant.

We would very much like to thank the reviewer for this question as it led us to a series of important analyses and reanalyses. The first, we requantified chromocenter number for two reasons. 1) Based on review of the data, it appears our previous chromocenter counts inadvertently included PMSC as chromocenters in the wildtype thus our initial counts had elevated numbers of wildtype round spermatids with more than one chromocenter. To correct for this, we used a more stringent requirement for DAPI intensity in a focus as PMSC generally has lower DAPI intensity than observed in chromocenters. This was performed using a more precise imaging system which was not available during our initial analysis. 2) our previous analysis failed to take into account tubule stage, thus was uninformative regarding whether the chromocenter phenotype was progressive or set in the round spermatid population. This new analysis revealed that chromocenter number was relatively fixed in mutant round spermatids, with 75 to 80% of the population having two or more chromocenters throughout their development while less than 15% of wildtype round spermatids showed the same. The outcome of this analysis is included in newly added Fig. 1C and is summarized in text from the manuscript below (in blue).

We next asked when during round spermatid development chromocenter number increases in *Adad2* mutant round spermatids via chromocenter quantification per round spermatid as a function of stage (Fig. 1C). As *Adad2* mutant tubules do not contain the full complement of post-meiotic cells normally used for staging, we relied instead on the immunofluorescent staining pattern of SYCP3 to aid in stage identification (SF 1A). This analysis demonstrated chromocenter defects were observed in the earliest population of round spermatids and, further, they did not increase through round spermatid development suggesting heterochromatin abnormalities in mutant spermatids arise prior to post-meiotic differentiation.

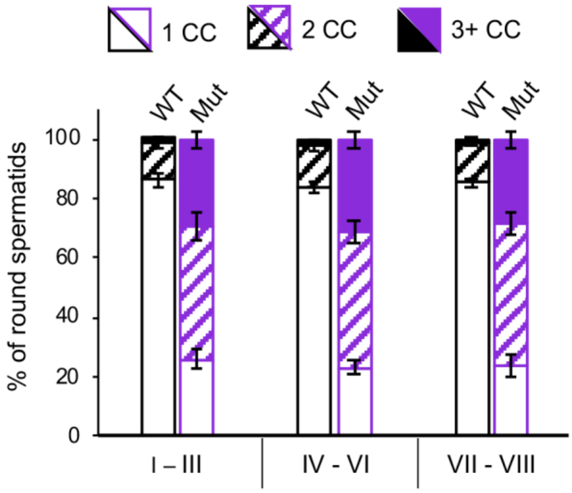

This rather surprising finding led us to re-examine our apoptosis data, which showed a bimodal distribution of apoptotic post-meiotic cells (new supplemental figure 2B) with tubules containing both round spermatids and the relatively rare elongating spermatids showing either no TUNEL+ cells or many TUNEL+ cells suggesting differences in apoptosis across the round spermatid population. To determine when apoptosis was occurring in round spermatids, we then quantified round spermatid number as a function of stage and determined round spermatid loss initiated in the earliest stages of development suggesting round spermatid loss occurred very early in their development. Text to this effect has been added to the results and can be found below in blue.

© 2022. Published by The Company of Biologists under the terms of the Creative Commons Attribution License (https://creativecommons.org/licenses/by/4.0/).

Lastly, we quantified spermatid apoptosis in *Adad2* mutants via TUNEL assay in adult testes (SF 2A-B). For both round spermatids in stages I through VIII and elongating spermatids in stages IX through XII, this analysis showed a bimodal distribution with roughly half of the mutant tubules containing many TUNEL-positive cells and the remaining having none suggesting spermatid apoptosis was occurring in distinct spermatid populations. To define which round spermatid population was undergoing apoptosis, round spermatids per tubule were quantified as a function of stage in adult testes (SF 2C). This analysis demonstrated an early (stage I-III) reduction of round spermatids in mutant testes relative to wildtype which held constant throughout the remainder of development. Together, these results imply an early loss of round spermatids at or just after the transition out of meiosis. This conclusion is further supported by normal levels of TUNEL-positive spermatocytes in mutant adults (SF 2D), which confirmed increased apoptotic germ cells were limited to post-meiotic spermatids.

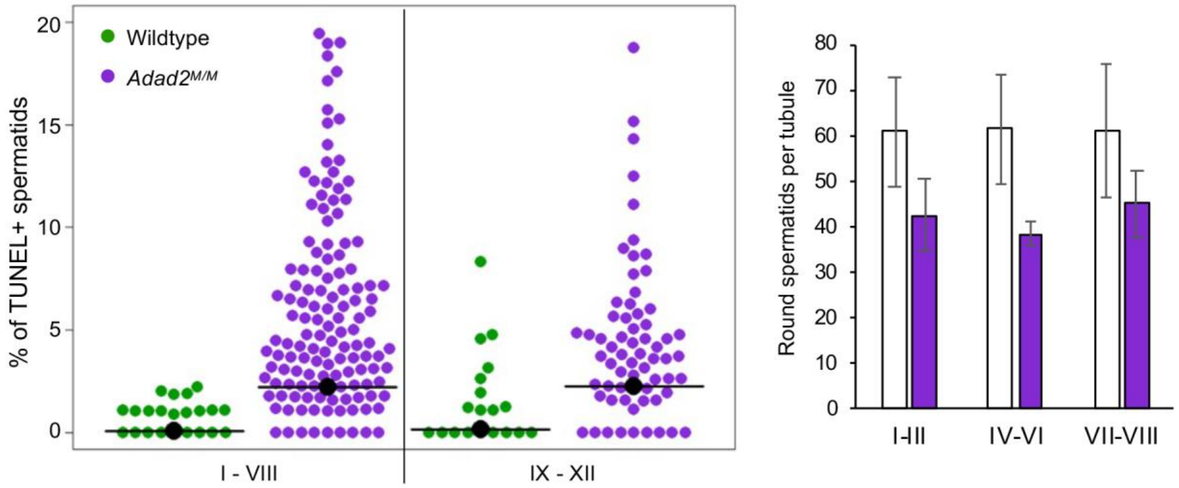

Additionally, discussion of this cell loss and potential mechanisms has been added to the discussion (see below in blue)

While the underlying molecular driver of the *Adad2* phenotype likely arises during mid to late pachytene, on the physiological level ADAD2 loss leads to significant reduction in post- meiotic germ cells (Chukrallah, et al, 2020). We propose cell loss occurs over two phases, the first at or just after the transition from meiotic spermatocyte to round spermatid and the second during the round spermatid to elongating spermatid transition. These results demonstrate that round spermatid development per se is not heavily reliant on proper heterochromatin distribution while cell type transitions involving chromatin rearrangements such as completion of meiosis and the histone to protamine transition are, further supporting the notion that abnormal heterochromatin is the primary driver of the *Adad2* mutant phenotype.

2. Fig. 1C. By comparing the WB analysis with quantification, it seems not to be a match between the image and the graph. HP1α expression is much stronger in Adad2M/M mice, than HP1ß and HP1γ. This is not the case in the graph at the side. Please, use images that are representative of quantification.

Western blot quantification was done using ImageJ on the provided images. Since ImageJ takes into account background signal within the quantified lane the relatively high background in the HP1a lanes results in an overall lower measurement of specific signal. Notation to this affect has been added to the methods.

3. Row 132. To conclude that mutant round spermatids truly have an altered chromatin state, you must proof it using enriched populations. Purified fractions of Rs can be obtained by either centrifugal elutriation, FACS sorting or STA-PUT velocity sedimentation. The latter allows isolation of purified fractions also from testis of a single mouse.

© 2022. Published by The Company of Biologists under the terms of the Creative Commons Attribution License (https://creativecommons.org/licenses/by/4.0/).

We very much appreciated this suggestion, which was also mentioned by another reviewer. To this end, we leveraged a small-scale STA-PUT method to isolate spermatocytes and round spermatids. These new analyses have been included as additional panels in Figure 1D and 1E (see reviewer 1 – point 2) and the enrichment values reported in Supplemental Figure 1B. As anticipated, these analyses demonstrated increased heterochromatin in both spermatocyte and round spermatids along with reduction of the euchromatin mark H3K4me2. In support of this, the expected pattern of increased HP1α, but not HP1β or HP1γ was also observed. Text to this effect has been added to the relevant results section.

4. Row 214. There is NO SF3A-C in the manuscript. SF3A-C and SF5 are identical.

We apologize for the error leading to this issue. We have taken special pains to ensure correct file uploads for this revision.

5. Row 2019. This mean that you cannot exclude that the Adad2-null phenotype is ascribed to deregulation of other protein factors. There is not much mention on that, especially in the discussion. Authors logically focus on MDC1 but they should discuss data in a broader context.

We entirely agree with this assessment and have added a paragraph discussing other likely players in the Adad2 phenotype to the discussion. The text of this can be found below in blue.

In this report, we focus on the impact of ADAD2 on DNA damage response proteins. However, it is important to note that ADAD2 likely regulates other important aspects of germ cell biology. Of particular interest is our observation both translation and ribosome assembly factors along with RNA processing proteins are encoded by transcripts with altered ribosome association of in *Adad2* mutants. Given the germ cell's tight reliance on post-transcriptional regulation (Braun et al., 1989; Kleene et al., 1984) it seems likely these mechanisms may be driving additional biologies not explained by abnormal DDR.

6. Fig. 3B. Staging is not convincing. Please, explain in detail how stages IV, VII and IX are univocally identified, in absence of normal germ cells association (mutant) and of stage-specific markers. Since γH2ax pattern is qualitatively not altered in the mutant, one way could be to use it to stage tubules in association with SYCP3 (see for instance supplementary material in Di Giacomo et al., Mol Cell 2013. DOI 10.1016/j.molcel.2013.04.026).

We apologize for not making our staging criteria clearer in the document. We do, in fact, rely on SYCP3 staining for staging throughout though we failed to explicitly note that in our previous draft. To resolve this, we have added a detailed description of staging criteria to the methods. Additionally, a new supplemental figure 1A has been added to aid this description.

7. Row 226. Figure 3C (stage VII) is not representative of what is stated in the text. Intensity of MDC1 in the sex body at stage VII is as low as that in stage XII. Moreover, stage XII is supposed to contain zygotene-stage spermatocytes, not late pachytene stage cells with sex body. Thus, you are not looking at the right cell type. A detailed analysis of MDC1 in mutat is lacking. Please, show what the pattern of MDC1 is, using chromosome spreads. Show MDC1 localization, level and spreading over the sex chromatin.

Unfortunately, MDC1 staining on our cell spreads is not technically feasible, which is why we focus on using immunofluorescence to define cell stage. We have repeated this analysis multiple times and always observe a similar pattern of MDC1 loss in the sex body of late pachytene spermatocytes and beyond (defined in tissue as spermatocytes observed in stage IX and beyond). Our identification of stage XII tubules is based on the criteria set out in Russell, et al. which states observation of any cells in metaphase, anaphase, or telophase of meiosis I defines the stage. In each case, we have identified by asterisk cells in this phase of meiosis to ensure clear identification of the stage. We additionally note, due to the rapid nature of these meiotic phases; tubules containing these cells nearly always contain diplotene spermatocytes as well. Thus, metaphase, anaphase, and telophase cells represent a tractable identification criterion for

© 2022. Published by The Company of Biologists under the terms of the Creative Commons Attribution License (https://creativecommons.org/licenses/by/4.0/).

diplotene spermatocytes, which still contain an intact sex body. We have added text to this effect to the figure legend. Lastly, we have replaced the image shown for the wildtype stage XII as cell identification was difficult in that particular panel.

8. Fig. 4B. Images are NOT representative of what shown in the graph. Levels of ATR in mid-pachynema in the mutant is much higher than what shown in the graph. Moreover, in the graph on abscissa you wrote “autosomal ATR”. In principle it is correct if you refer to cells where XY chromosomes are identified, and you have excluded ATR-associated to XY from the measure. In the mutant, this is likely doable in cells from late-pachytene onward, when XY are visible, not in early to mid-pachytene stage, where XY-associated ATR cannot be distinguished from that associated with the autosomes. Please, explain in detail how measurement of ATR signal onto autosomes was performed, excluding ATR signal associated to XY chromosomes.

We apologize for the confusion regarding our quantification measures, which has been extensively edited to clarify. In short, quantification is not on the individual level, but rather as a population whole. To that end, graphs represent the frequency of a given staining pattern within a population. Images represent localization patterns under or overrepresented in mutants relative to wildtype. Description of this has been included in the results, figure legends, as well as the methods. As for identification of XY versus autosome localization, we utilized the XY identification method outlined in Luo et al, 2015 developed in the Jeremy Wang laboratory which relies on both SYCP3 pattern on the axes as well as morphology of the condensing X and Y for stage identification. This has been fully described in the methods.

9. Row 255. There is not persistent association of ATR in late-pachytene stage cells, while phosphorylatin of H2AX persists. How do you reconcile this result with your interpretation? Authors should check persistence of DSBs using one additional DSB repair marker such as DMC1. According to Testa et al., JCS 2018 (DOI 10.1242/jcs.214411), a lack of MDC1 reduces the stringency of the recombination- dependent checkpoint and it allows progression to mid-pachynema of cells with unrepaired DSBs (i.e. with greater number of DMC1 foci). Thus, the increased γH2AX level at late-pachynema might be linked to the persistence of DSBs intermediates that are not marked by RPA.

Due to technical restraints from our cell spread protocol, we are unable to stain for DMC1. As a result, we cannot entirely confirm Spo11-induced breaks are resolved in mutant spermatocytes. That being said, several lines of evidence support the notion they are. First, as previously shown, RPA foci decrease normally in mutants as spermatocytes develop. Secondly, as we newly show in supplemental figure 6D, neither Rad9 nor Ku80 are increased in Adad2 mutants, which would be expected as persistent DSBs should lead to activation of DDR pathways. Given we observe normal ATR and gH2AX at mid-pachytene and prior and MDC1 levels are normal until late pachytene (stage VII and beyond), we conclude the recombination checkpoint is intact (and inactive) in Adad2 mutants suggesting Spo11-induced breaks are resolved properly. A statement to this effect has been added to both the results.

To the point of MDC1-regulated checkpoints, we would especially like to thank the reviewer for pointing out the Testa report which we read with interest. As a result of this report, we have initiated studies to examine whether MDC1 modulates an additional checkpoint in late meiosis. These studies are based on our observation of late meiotic or early post-meiotic cell loss in Adad2 mutants (see newly added data in supplemental figure 2B and 2C). However, stringent testing of this hypothesis requires additional studies well beyond the scope of this report are required. These studies are underway, and we anticipate reporting on them when complete.
Brief discussion of this exciting possibility is included in the discussion.

Minor concerns:

1. Please provide a table with all primary and secondary antibodies used in the study, including source, catalog number, dilutions and application.

© 2022. Published by The Company of Biologists under the terms of the Creative Commons Attribution License (https://creativecommons.org/licenses/by/4.0/).

An antibody table has been provided. Please see Supplemental Figure 8A.

2. Row 18. The first demonstration that failure in XY body formation results in meiotic cells arrest comes from the studies on the role of histone H2AFX in meiosis. The reference below should thus be included:
    a. Fernandez-Capetillo et al., H2AX is required for chromatin remodeling and inactivation of sex chromosomes in male mouse meiosis, Dev Cell, 2003. PMID 12689589

This reference has been added.

3. Row 43. In addition to MSCI, MDC1 is also involved in controlling recombination (MLH3 assembly) and the activation of the recombination-dependent checkpoint. The following reference should be included in the list:
    a. Testa et al., H2AFX and MDC1 promote maintenance of genomic integrity in male germ cells, JCS 2018. DOI 10.1242/jcs.214411

This reference, and brief discussion there of, has been added to the introduction and the findings have also been included within the discussion.

4. Fig.1B. It would be good to have Rs identified by H1t staining. Alternatively, please indicate whether you are looking at the same stage of the epithelial cell cycle in wt Vs Adad2M/M, and identify the stage.

Images from Figure 1B were stage matched based on spermatocyte morphology, which indicated they are stage IV to VI. This has been indicated in the figure legend.

5. Row 115. 1B should be 1C

This has been corrected.

6. Fig. 1C. Statistical significance of HP1□ expression in adult is limited. This is likely due to the fact you are using total testis extracts. To increase significance of Hp1□ expression, I suggest performing western blotting from isolated fractions of Rs.

As discussed above, all HP1 protein and histone marks reported have now been assessed in individual cell fractions. Please see additional panels in Figure 1D and 1E.

7. Fig. 1D. Again, to increase significance in methylation pattern changes, I suggest performing western blotting from isolated fractions of round spermatids.

See above.

8. Row 145. It is not clear to me how data shown in S1A have been obtained. Have you isolated and analyzed wild type cells, or you just assigned DE genes to each testis cell category accordingly to data in literature?

In this case, cell assignment is based on expression in a publicly available dataset. Details of the analysis can be found in the methods and a brief clarification added to the figure legend.

9. Fig. S1E-F. How have you identified stages of the epithelial cell cycle? It is very hard to do it by just using DAPI stained sections. Lack of post meiotic germ cells in the mutant makes identification of stages even more difficult. Please explain procedure in detail.

A detailed description of staging criteria has been added to the methods and the staging patterns described in a new supplemental figure (1A).

© 2022. Published by The Company of Biologists under the terms of the Creative Commons Attribution License (https://creativecommons.org/licenses/by/4.0/).

10. Fig. 7C. Please provide evidence of antibody staining specificity in elongated spermatids by blocking with a TNP1 peptide and showing in supplementary the staining pattern with just secondary antibodies.

This antibody is commercial and has been previously validated (see Supplemental Figure 8A). We have, however provided images of staining with secondary antibodies only as inset in the main figure.

Second decision letter

MS ID#: JOCES/2021/259196

MS TITLE: ADAD2 regulates heterochromatin in meiotic and post-meiotic male germ cells via translation of MDC1

AUTHORS: Lauren G Chukrallah, Aditi Badrinath, Gabrielle G Vittor, and Elizabeth M Snyder
ARTICLE TYPE: Research Article

I am happy to tell you that your manuscript has been accepted for publication in Journal of Cell Science, pending standard ethics checks.

Reviewer 1

Advance summary and potential significance to field

The authors did a wonderful job addressing the comments and making the story richer with more data.
Overall is a very nice paper.

Comments for the author

I am satisfied with this revision.

Reviewer 3

Advance summary and potential significance to field

In this manuscript Lauren G. Chukrallah et al., describes how ADAD2 regulates meiotic and post-meiotic heterochromatin via MDC1. The authors demonstrate that maintenance of MSCI that is established early in meiosis, is key to proper differentiation of post-meiotc germ cells.
This is a very interesting work that answers a relevant biological question in the field. The manuscript is well written, and most conclusions straight forward and supported by the experimental results. There is no doubt it deserves publication. Response of the authors are satisfactory, thus I recommend publication.

Comments for the author

It is my opinion that after the first round of revision the manuscript can be accepted for publication on JCS

© 2022. Published by The Company of Biologists under the terms of the Creative Commons Attribution License (https://creativecommons.org/licenses/by/4.0/).
